# Predicting eating disorder and anxiety symptoms using disorder-specific and transdiagnostic polygenic scores for anorexia nervosa and obsessive-compulsive disorder

**DOI:** 10.1017/S0033291721005079

**Published:** 2023-05

**Authors:** Zeynep Yilmaz, Katherine Schaumberg, Matthew Halvorsen, Erica L. Goodman, Leigh C. Brosof, James J. Crowley, Anorexia Nervosa Genetics Initiative, Eating Disorders Working Group of the Psychiatric Genomics Consortium, Tourette Syndrome/Obsessive-Compulsive Disorder Working Group of the Psychiatric Genomics Consortium, Carol A. Mathews, Manuel Mattheisen, Gerome Breen, Cynthia M. Bulik, Nadia Micali, Stephanie C. Zerwas

**Affiliations:** 1 National Centre for Register-based Research, Aarhus BSS, Aarhus University, Aarhus, Denmark; 2 Department of Psychiatry, University of North Carolina, Chapel Hill, NC, USA; 3 Department of Genetics, University of North Carolina, Chapel Hill, NC, USA; 4 Department of Medical Epidemiology and Biostatistics, Karolinska Institutet, Stockholm, Sweden; 5 Department of Psychiatry, University of Wisconsin, Madison, WI, USA; 6 Department of Psychology, University of North Dakota, Grand Forks, ND, USA; 7 Department of Psychological and Brain Sciences, University of Louisville, Louisville, KY, USA; 8 Department of Clinical Neuroscience, Karolinska Institutet, Stockholm, Sweden; 9 Department of Psychiatry, Genetics Institute, University of Florida, Gainesville, FL, USA; 10 Department of Biomedicine, Aarhus University, Høegh-Guldbergs Gade 10, Aarhus, Denmark; 11 The Lundbeck Foundation Initiative of Integrative Psychiatric Research (iPSYCH), Aarhus, Denmark; 12 Department of Psychiatry, Psychosomatics and Psychotherapy, University of Würzburg, Würzburg, Germany; 13 Institute of Psychiatry, Psychology and Neuroscience, MRC Social, Genetic and Developmental Psychiatry (SGDP) Centre, King's College London, London, UK; 14 National Institute for Health Research Biomedical Research Centre, South London and Maudsley National Health Service Trust, London, UK; 15 Department of Nutrition, Gillings School of Global Public Health, University of North Carolina, Chapel Hill, NC, USA; 16 Department of Psychiatry, Faculty of Medicine, University of Geneva, HUG, Geneva, Switzerland; 17 Institute of Child Health, University College London, London, UK; 18 Department of Paediatrics, Gynecology and Obstetrics, Faculty of Medicine, University of Geneva, HUG, Geneva, Switzerland

**Keywords:** Eating disorders, obsesive-compulsive disorder, polygenic scores, anxiety, developmental cohort

## Abstract

**Background:**

Clinical, epidemiological, and genetic findings support an overlap between eating disorders, obsessive-compulsive disorder (OCD), and anxiety symptoms. However, little research has examined the role of genetics in the expression of underlying phenotypes. We investigated whether the anorexia nervosa (AN), OCD, or AN/OCD transdiagnostic polygenic scores (PGS) predict eating disorder, OCD, and anxiety symptoms in a large developmental cohort in a sex-specific manner.

**Methods:**

Using summary statistics from Psychiatric Genomics Consortium AN and OCD genome-wide association studies, we conducted an AN/OCD transdiagnostic genome-wide association meta-analysis. We then calculated AN, OCD, and AN/OCD PGS in participants from the Avon Longitudinal Study of Parents and Children to predict eating disorder, OCD, and anxiety symptoms, stratified by sex (combined *N* = 3212–5369 per phenotype).

**Results:**

The PGS prediction of eating disorder, OCD, and anxiety phenotypes differed between sexes, although effect sizes were small. AN and AN/OCD PGS played a more prominent role in predicting eating disorder and anxiety risk than OCD PGS, especially in girls. AN/OCD PGS provided a small boost over AN PGS in the prediction of some anxiety symptoms. All three PGS predicted higher compulsive exercise across different developmental timepoints [*β =* 0.03 (s.e. = 0.01) for AN and AN/OCD PGS at age 14; *β =* 0.05 (s.e. = 0.02) for OCD PGS at age 16] in girls.

**Conclusions:**

Compulsive exercise may have a transdiagnostic genetic etiology, and AN genetic risk may play a role in the presence of anxiety symptoms. Converging with prior twin literature, our results also suggest that some of the contribution of genetic risk may be sex-specific.

## Introduction

Eating disorders and obsessive-compulsive disorder (OCD) are serious psychiatric conditions with high social, psychological, and physical impact (American Psychiatric Association, 2013; Keshaviah et al., 2014; World Health Organization, 2008). Clinical, epidemiological, and genetic findings support an overlap between eating disorders and anxiety disorders, particularly anorexia nervosa (AN), and OCD (Anttila et al., 2018; Cederlof et al., 2015; du Toit, van Kradenburg, Niehaus, & Stein, 2001; Godart, Flament, Perdereau, & Jeammet, 2002; Kaye, Bulik, Thornton, Barbarich, & Masters, 2004; Lilenfeld et al., 1998; Meier et al., 2015; Rubenstein, Pigott, L'Heureux, Hill, & Murphy, 1992; Strober, Freeman, Lampert, & Diamond, 2007; Swinbourne & Touyz, 2007; Watson et al., 2019; Yilmaz et al., 2020). Whilst research on eating disorders and OCD comorbidity has primarily focused on diagnoses, many symptoms and behaviors are common to both diagnoses, spanning diagnostic categories, and their presence often precedes disorder onset (Nolen-Hoeksema & Watkins, 2011; Stice, 2016). Little research has examined these associations – or symptom phenotypes – in a developmental context. Premorbid OCD symptoms and anxiety disorders or symptoms are common in patients with AN (Cederlof et al., 2015; Schaumberg et al., 2019). Childhood anxiety may precede eating disorder symptoms and AN in adolescence (Schaumberg et al., 2019), and shared genetic and environmental influences play a role in anxiety and disordered eating symptoms (Silberg & Bulik, 2005). Though no longer classified as an anxiety disorder (American Psychiatric Association, 2013), OCD is highly comorbid with anxiety disorders and includes anxiety symptoms, especially in children (Anagnostopoulos et al., 2016). An improved understanding of the overlap among eating disorders, OCD, and intermediate phenotypes such as anxiety symptoms could aid in conceptualizing mechanisms and processes contributing to the clinical and genetic overlap among these disorders. Additionally, symptom dimensions may transmute over development, shifting from childhood obsessive-compulsive symptoms to adolescent eating disorders (Anderluh, Tchanturia, Rabe-Hesketh, & Treasure, 2003; Micali et al., 2011) and vice versa. Thus, shared and unique risk factors may contribute to the symptoms of OCD and eating disorders across development.

Genome-wide association studies (GWAS) of AN (Watson et al., 2019) and OCD [International Obsessive Compulsive Disorder Foundation Genetics Collaborative (IOCDF-GC) & OCD Collaborative Genetics Association Studies (OCGAS), 2018] have provided important insights into the highly polygenic architecture of these disorders and their positive genetic correlation (Watson et al., 2019). Application of polygenic scores (PGS) – the weighted sum of common risk variants per individual – examine the genetic architecture of complex traits using evidence for association from variants below the stringent threshold for genome-wide significance (Wray et al., 2014). The use of PGS has been validated across psychiatric diagnoses and symptom-level measures (Axelrud et al., 2018; Cross-Disorder Group of the Psychiatric Genomics Consortium et al., 2013; Lee et al., 2013; Mistry, Harrison, Smith, Escott-Price, & Zammit, 2018; Psychiatric GWAS Consortium Bipolar Disorder Working Group, 2011; Ripke et al., 2013, 2014), demonstrating that genetic variants associated with risk are often shared across diagnostic categories (Mistry et al., 2018). Moreover, transdiagnostic PGS (determined by either AN or OCD case status) of genetically correlated disorders may enhance predictive power for either disorder (Maier et al., 2015).

Sex differences in the prevalence and presentation of eating disorders, anxiety disorders, and OCD warrant sex-specific examination of risk factors. While the majority of AN cases are female (Hudson, Hiripi, Pope, & Kessler, 2007; Swanson, Crow, Le Grange, Swendsen, & Merikangas, 2011), AN in males often has an earlier age of onset and is likely to be more severe (El Ghoch, Calugi, Milanese, Bazzani, & Dalle Grave, 2017; Kinasz, Accurso, Kass, & Le Grange, 2016; Voderholzer et al., 2019). Similarly, the lifetime prevalence of eating disorders is much higher in females than males (Hudson et al., 2007; Swanson et al., 2011), possibly with the exception of subthreshold binge eating (Hudson et al., 2007). Furthermore, the twin literature has reported differences in the heritability estimates for disordered eating in boys and girls (Klump et al., 2012). The lifetime prevalence of anxiety disorders is up to 60% higher in women than in men (Kessler, Chiu, Demler, Merikangas, & Walters, 2005). In the case of OCD, childhood onset is more common among males and adolescent onset is more common among females (Ruscio, Stein, Chiu, & Kessler, 2010). Importantly, sex differences in the presentation of symptoms such as restraint and weight and shape concern in eating disorders (Kinasz et al., 2016) and contamination/cleaning and sexual/religious symptoms in OCD (Torresan et al., 2013) have also been reported. Given these discrepancies, we could expect: (a) notable sex differences in the role of genetic risk and eating disorders, OCD, and anxiety symptom phenotypes; and (b) that genetic risk may be more impactful and predictive for boys, especially in the case of eating disorders.

This study examined whether the AN, OCD, or AN/OCD PGS predicts eating disorders, OCD, and anxiety symptom dimensions or diagnoses using a developmental framework in male and female participants from a population-based cohort. Our main hypothesis was that AN/OCD PGS would demonstrate better statistical power than AN or OCD PGS, and the transdiagnostic PGS would evidence the most benefit compared with single-trait PGS when predicting intermediate phenotypes shared across the two disorders, such as generalized anxiety or worrying. We also hypothesized that symptom dimensions specific to each disorder would be predicted by disorder-specific PGS (e.g. thin ideal internalization by AN, or symmetry/checking behavior by OCD). Importantly, in light of the differences in lifetime prevalence and/or age of onset of eating disorders, OCD, and anxiety disorders between sexes, we hypothesized that there would be sex-specific differences in the prediction of AN, OCD, and AN/OCD PGS, and high AN genetic risk would play a larger role in predicting eating disorder symptoms in boys than girls.

## Methods

### Participants

The Avon Longitudinal Study of Parents and Children (ALSPAC) is a longitudinal, population-based study of women and their children (Boyd et al., 2013). All pregnant women living in Avon, United Kingdom who were expected to deliver between 1 April 1991 and 31 December 1992 were invited to participate. Children from 14 541 pregnancies were enrolled, 13 988 of whom were alive at one year. An additional 713 children were enrolled at or after age 7 (Boyd et al., 2013). The study website contains details of all the data that are available through a fully searchable data dictionary and variable search tool (http://www.bristol.ac.uk/alspac/researchers/our-data/). Ethical approval was obtained from the ALSPAC Ethics and Law Committee and the Local Research Ethics Committees. Briefly, informed consent for the use of data collected via questionnaires and clinics was obtained from participants following the recommendations of the ALSPAC Ethics and Law Committee at the time. Mothers provided written consent for the participation of their children, and children were invited to give assent whenever it was appropriate. Study participants have the right to withdraw their consent for elements of the study or from the study entirely at any time. Full details of the ALSPAC consent procedures are available on the study website (http://www.bristol.ac.uk/alspac/researchers/research-ethics/).

For genetic analyses, we used post-quality control (QC) dosage files for 7977 unrelated participants (Martin, Hamshere, Stergiakouli, O'Donovan, & Thapar, 2014; Paternoster et al., 2012), 7779 of whom passed additional QC performed as a part of this study (3787 girls and 3992 boys; see online Supplementary Information). The final number of participants with genotype and at least one phenotype information was 3270 girls and 3297 boys.

### Measures

Table 1 provides a list of all measures, assessment timepoints, and methods of administration. Measures assessing psychopathology at younger ages (before age 14) were primarily assessed via parent-report. Those assessing psychopathology during adolescence (age 14 or older) were primarily assessed via self-report.

**Table 1.** Eating disorder, obsessive-compulsive disorder, and anxiety diagnostic and symptom-based constructs

Age(s)	Construct	Scale	Report
*Eating disorder*			
10	Body image distortion	Stunkard Figure Rating Scale	Self
14	Fear of weight gain	Youth Risk Behavior Surveillance System Questionnaire	Self
	Pressure to lose weight	Perceived Sociocultural Pressure Scale	Self
	Restraint	Dutch Eating Behavior Questionnaire	Self
	Emotional eating		
	External eating		
	Thin-ideal internalization	Ideal-Body Stereotype Scale-Revised	Self
	Body dissatisfaction	Satisfaction and Dissatisfaction With Body Parts Scale	Self
	Weight and shape concern	McKnight Risk Factor Survey	Self
14 and 16	Anorexia nervosa	Youth Risk Behavior Surveillance System Questionnaire	Self
	Bulimia nervosa or subthreshold bulimia nervosa		
	Binge-eating disorder or subthreshold binge-eating disorder		
	Eating disorders not otherwise specified or purging disorder		
	Any threshold/subthreshold eating disorder		
	Fasting		
	Purging		
	Binge eating		
	Compulsive exercise		
*Obsessive-compulsive disorder*			
10 and 13	Obsessive-compulsive disorder	Development and Wellbeing Assessment	Parent
	Obsessive-compulsive disorder latent factor symmetry		
	Obsessive-compulsive disorder latent factor dirt/germs		
*Anxiety*			
7	Separation anxiety	Development and Wellbeing Assessment	Parent
10 and 13	Social phobia		
	Latent factor physical anxiety		
	Latent factor worrying		
	Latent factor social phobia		
13	Generalized anxiety disorder	Development and Wellbeing Assessment	Parent
15		Development and Wellbeing Assessment	Self

Eating disorder symptoms for the previous year were evaluated at ages 14 and 16 using questions adapted from the Youth Risk Behavior Surveillance System Questionnaire (Kann et al., 1996), validated in a population-based study of adolescents (Field, Taylor, Celio, & Colditz, 2004). Binge-eating, purging, fasting, and compulsive exercise were characterized and categorized as described previously (Micali, Daniel, Ploubidis, & De Stavola, 2018; Micali et al., 2015) (online Supplementary Information). Eating disorder diagnoses at ages 14 and 16 were derived using DSM-5 criteria (American Psychiatric Association, 2013) as detailed in a previous publication by our group (Schaumberg et al., 2019). Eating disorder cognitions, including body image distortion, emotional eating, external eating, body dissatisfaction, thin ideal internalization, dietary restraint, weight concern, and shape concern, were assessed by validated, age-appropriate self-report measurements (online Supplementary Information).

OCD and anxiety symptoms at age 7, 10, 13, and 15 were collected using the Development and Wellbeing Assessment (DAWBA; online Supplementary Information) (Goodman, Ford, Richards, Gatward, & Meltzer, 2000; Goodman, Heiervang, Collishaw, & Goodman, 2011). Probabilities of anxiety disorder diagnoses at ages 7 (specific phobia and separation anxiety), 10 (OCD), 13 (OCD, social phobia, and generalized anxiety disorder), and 15 (generalized anxiety disorder) were determined using computer-generated DAWBA band variables (Goodman et al., 2011), which assign the probability of the participant meeting DSM-IV criteria for an anxiety disorder. We defined likely cases as those where likelihood of case status based on response pattern was ⩾50%. We also defined five latent OCD or anxiety factors for ages 10 and 13: (1) OCD-symmetry; (2) OCD-dirt/germs; (3) physical anxiety; (4) worrying; and (5) social phobia (Schaumberg et al., 2019) (online Supplementary Information).

### Data analysis

We calculated AN, OCD, and AN/OCD PGS to predict 27 eating disorder, six OCD, and 11 anxiety phenotypes in the ALSPAC target sample using PRS-CS (Ge, Chen, Ni, Feng, & Smoller, 2019). AN PGS was constructed using the Anorexia Nervosa Genetics Initiative & Psychiatric Genomics Consortium (PGC) Eating Disorder Working Group Freeze 2 AN GWAS (Watson et al., 2019), and OCD PGS was calculated using the Freeze 1 PGC OCD GWAS [International Obsessive Compulsive Disorder Foundation Genetics Collaborative (IOCDF-GC) & OCD Collaborative Genetics Association Studies (OCGAS), 2018]. The AN/OCD summary statistics file was obtained from a GWAS meta-analysis of the AN and OCD datasets (see online Supplementary Information, Table S1, and Fig. S1). All of the discovery samples and the ALSPAC target sample included in our analysis were of European ancestry, determined using genomic ancestry principal components through comparison with a European ancestry (CEU) reference panel. We examined how well each of the eating disorder, OCD, and anxiety symptom phenotypes were predicted by: (1) AN; (2) OCD; and (3) AN/OCD PGS in girls and boys separately to elucidate whether sex-specific differences existed. Additional results for the combined sample with and without sex as a covariate are summarized in online Supplementary Tables S3 and S4. Due to insufficient power, only binary phenotypes with ⩾50 cases are reported.

## Results

### Eating disorder symptom phenotypes and diagnoses

In girls, AN PGS predicted eating disorders not otherwise specified/purging disorder at age 14 [*ß* = 0.1130 (0.0552), *p* = 0.041], presence of a threshold or subthreshold eating disorder at age 14 [*ß* = 0.1214 (0.0498), *p* = 0.015], and compulsive exercise at age 14 [*ß* = 0.0336 (0.0143), *p* = 0.019] (Table 2). OCD PGS predicted thin ideal internalization at age 14 [*ß* = 0.1264 (0.0487), *p* = 0.010] and compulsive exercise at age 16 [*ß* = 0.0535 (0.0240), *p* = 0.025]. AN/OCD PGS predicted pressure to lose weight at age 14 [*ß* = 0.0839 (0.0423), *p* = 0.047], the presence of a threshold or subthreshold eating disorder at age 14 [*ß* = 0.1146 (0.0493), *p* = 0.020], fasting at age 14 [*ß* = 0.0148 (0.0064), *p* = 0.020], and compulsive exercise at age 14 [*ß* = 0.0287 (0.0142), *p* = 0.043].

**Table 2.** Prediction of eating disorder, obsessive-compulsive disorder, and anxiety symptom dimensions and diagnoses using polygenic scores in girls^a^

Phenotype	Sample size	PGS	*ß*	s.e.	Test statistic^b^	*P*
*Eating disorder symptom dimensions and diagnoses*						
Body image distortion at age 10	2512	AN	−0.0039	0.0138	−0.283	0.777
		OCD	0.0078	0.0133	0.587	0.557
		AN/OCD	−0.0052	0.0137	−0.381	0.703
Fear of weight gain at age 14	2277	AN	0.0200	0.0164	1.222	0.222
		OCD	0.0099	0.0159	0.624	0.533
		AN/OCD	0.0242	0.0162	1.489	0.137
Pressure to lose weight at age 14	2263	AN	0.0813	0.0428	1.900	0.057
		OCD	0.0201	0.0414	0.486	0.627
		AN/OCD	0.0839	0.0423	1.984	0.047*
Restraint at age 14	2257	AN	0.0478	0.0269	1.774	0.076
		OCD	0.0091	0.0259	0.350	0.726
		AN/OCD	0.0501	0.0267	1.880	0.060
Emotional eating at age 14	2167	AN	−0.0006	−0.1294	0.005	0.996
		OCD	0.0361	0.1248	0.289	0.772
		AN/OCD	−0.0447	0.1279	−0.349	0.727
External eating at age 14	2068	AN	0.0621	0.0732	0.849	0.396
		OCD	0.0003	0.0709	0.004	0.990
		AN/OCD	0.0322	0.0726	0.444	0.657
Thin ideal internalization at age 14	2283	AN	0.0755	0.0505	1.495	0.135
		OCD	0.1264	0.0487	2.593	0.010*
		AN/OCD	0.0796	0.0500	1.592	0.112
Body dissatisfaction at age 14	2297	AN	0.2948	0.1677	1.758	0.079
		OCD	0.2531	0.1620	1.562	0.118
		AN/OCD	0.2899	0.1661	1.745	0.081
Weight and shape concern at age 14	2299	AN	0.0331	0.0417	0.794	0.428
		OCD	0.0392	0.0404	0.970	0.332
		AN/OCD	0.0481	0.0413	1.164	0.245
AN at age 14^c^	78 cases, 2242 controls	AN	0.1734	0.1180	1.470	0.142
		OCD	0.0624	0.1136	0.550	0.583
		AN/OCD	0.1845	0.1167	1.582	0.114
Bulimia nervosa or subthreshold bulimia nervosa at age 14^c^^,^^d^	44 cases, 2276 controls	AN	–	–	–	–
		OCD	–	–	–	–
		AN/OCD	–	–	–	–
Binge-eating disorder or subthreshold binge-eating disorder at age 14^c^^,^^d^	17 cases, 2303 controls	AN	–	–	–	–
		OCD	–	–	–	–
		AN/OCD	–	–	–	–
Eating disorders not otherwise specified or purging disorder at age 14^c^	419 cases, 1901 controls	AN	0.1130	0.0552	2.045	0.041*
		OCD	−0.0157	0.0534	−0.294	0.769
		AN/OCD	0.1026	0.0546	1.878	0.060
Any threshold/subthreshold eating disorder at age 14^c^	558 cases, 1762 controls	AN	0.1214	0.0498	2.439	0.015*
		OCD	−0.0003	0.0480	−0.007	0.994
		AN/OCD	0.1146	0.0493	2.327	0.020*
Fasting at age 14	2074	AN	0.0122	0.0064	1.897	0.058
		OCD	−0.0031	0.0062	−0.497	0.619
		AN/OCD	0.0148	0.0064	2.327	0.020*
Purging at age 14	2278	AN	0.0058	0.0068	0.851	0.395
		OCD	−0.0098	0.0066	−1.484	0.138
		AN/OCD	0.0041	0.0068	0.602	0.548
Binge eating at age 14	2289	AN	−0.0048	0.0100	−0.505	0.613
		OCD	0.0097	0.0092	1.053	0.292
		AN/OCD	−0.0015	0.0095	−0.154	0.877
Compulsive exercise at age 14	2240	AN	0.0336	0.0143	2.349	0.019*
		OCD	−0.0001	0.0138	0.006	0.996
		AN/OCD	0.0287	0.0142	2.023	0.043*
AN at age 16^c^	56 cases, 2035 controls	AN	−0.0151	0.1370	−0.110	0.912
		OCD	0.0785	0.1333	0.589	0.556
		AN/OCD	−0.0390	0.1351	−0.288	0.773
Bulimia nervosa at age 16^c^	121 cases, 1970 controls	AN	0.0623	0.0945	0.660	0.510
		OCD	−0.0112	0.0927	−0.121	0.904
		AN/OCD	0.0371	0.0934	0.398	0.691
Binge-eating disorder at age 16^c^^,^^d^	48 cases, 2043 controls	AN	–	–	–	–
		OCD	–	–	–	–
		AN/OCD	–	–	–	–
Eating disorders not otherwise specified or purging disorder at age 16^c^	843 cases, 1248 controls	AN	0.0455	0.0450	1.012	0.312
		OCD	0.0145	0.0440	0.329	0.742
		AN/OCD	0.0394	0.0444	0.887	0.375
Any threshold/subthreshold eating disorder at age 16^c^	1068 cases, 1023 controls	AN	0.0528	0.0447	1.195	0.232
		OCD	0.0441	0.0433	1.020	0.308
		AN/OCD	0.0394	0.0436	0.904	0.366
Fasting at age 16	1972	AN	−0.0040	0.0180	−0.220	0.826
		OCD	0.0066	0.0176	0.378	0.705
		AN/OCD	−0.0097	0.0178	−0.545	0.586
Purging at age 16	1990	AN	0.0133	0.0140	0.954	0.340
		OCD	0.0091	0.0136	0.667	0.505
		AN/OCD	0.0123	0.0138	0.892	0.372
Binge eating at age 16	1715	AN	0.0169	0.0183	0.923	0.356
		OCD	0.0323	0.0181	1.789	0.074
		AN/OCD	0.0149	0.0181	0.823	0.411
Compulsive exercise at age 16	1840	AN	0.0262	0.0248	1.058	0.290
		OCD	0.0535	0.0240	2.233	0.025*
		AN/OCD	0.0179	0.0245	0.731	0.465
*Obsessive-compulsive disorder symptom dimensions and diagnosis*						
OCD at age 10^c^^,^^d^	<5 cases	AN	–	–	–	–
		OCD	–	–	–	–
		AN/OCD	–	–	–	–
OCD latent factor – symmetry, checking at age 10	2590	AN	0.0037	0.0094	0.397	0.691
		OCD	0.0106	0.0090	1.173	0.241
		AN/OCD	0.0013	0.009	0.138	0.890
OCD latent factor – dirt/germs at age 10	2590	AN	−0.0002	0.0083	−0.027	0.979
		OCD	0.0127	0.0080	1.585	0.113
		AN/OCD	−0.0010	0.0083	−0.110	0.913
OCD at age 13^c^^,^^d^	<5 cases	AN	–	–	–	–
		OCD	–	–	–	–
		AN/OCD	–	–	–	–
OCD latent factor – symmetry, checking at age 13	2421	AN	−0.0208	0.0151	−1.381	0.167
		OCD	0.0183	0.0147	1.246	0.213
		AN/OCD	−0.0186	0.0150	−1.241	0.215
OCD latent factor – dirt/germs at age 13	2421	AN	0.0281	0.0129	2.175	0.030*
		OCD	−0.0157	0.0126	−1.248	0.212
		AN/OCD	0.0277	0.0128	2.160	0.031*
*Anxiety symptom dimensions and diagnoses*						
Separation anxiety at age 7^c^	68 cases, 2544 controls	AN	0.4342	0.1246	3.485	0.001*
		OCD	0.1147	0.1228	0.934	0.350
		AN/OCD	0.4868	0.1246	3.906	<0.001*
Specific phobia at age 7^c^	53 cases, 2564 controls	AN	0.2390	0.1406	1.699	0.089
		OCD	0.1661	0.1381	1.203	0.229
		AN/OCD	0.2519	0.1400	1.799	0.072
Latent factor – physical anxiety at age 10	2590	AN	0.0287	0.0147	1.953	0.051
		OCD	0.0150	0.0142	1.056	0.291
		AN/OCD	0.0196	0.0147	1.340	0.181
Latent factor – worrying at age 10	2590	AN	0.0078	0.0148	0.526	0.599
		OCD	0.0065	0.0143	0.450	0.652
		AN/OCD	0.0077	0.0148	0.520	0.603
Latent factor – social phobia at age 10	2590	AN	0.0114	0.0119	0.958	0.338
		OCD	0.0131	0.0115	1.139	0.255
		AN/OCD	0.0078	0.0119	0.655	0.513
Social phobia at age 13^c^^,^^d^	30 cases, 2396 controls	AN	–	–	–	–
		OCD	–	–	–	–
		AN/OCD	–	–	–	–
Generalized anxiety disorder at age 13^c^	100 cases, 2317 controls	AN	0.1078	0.1040	1.036	0.300
		OCD	0.1597	0.1019	1.567	0.117
		AN/OCD	0.1120	0.1029	1.088	0.276
Latent factor – physical anxiety at age 13	2421	AN	−0.0012	0.0132	−0.092	0.927
		OCD	0.0042	0.0129	0.325	0.746
		AN/OCD	−0.0032	0.0131	−0.241	0.810
Latent factor – worrying at age 13	2421	AN	0.0303	0.0165	1.832	0.067
		OCD	0.0019	0.0161	0.116	0.908
		AN/OCD	0.0334	0.0164	2.035	0.042*
Latent factor – social phobia at age 13	2421	AN	0.0306	0.0156	1.961	0.050
		OCD	0.0032	0.0152	0.209	0.834
		AN/OCD	0.0367	0.0155	2.374	0.018*
Generalized anxiety disorder at age 15^c^	154 cases, 1899 controls	AN	0.1188	0.0858	1.385	0.166
		OCD	0.0656	0.0835	0.786	0.432
		AN/OCD	0.0895	0.0850	1.054	0.292

Abbreviations: PGS, polygenic score; *ß*, standardized beta regression coefficient; s.e., standard error; AN, anorexia nervosa; OCD, obsessive-compulsive disorder; AN/OCD, anorexia nervosa/obsessive-compulsive transdiagnostic phenotype.

a Genomic principal components 1–5 were used as covariates to account for population stratification.

b We report *t*-values for continuous phenotypes and *z*-values for binary phenotypes.

c Binary phenotype.

d Due to insufficient statistical power, any binary measure with less than 50 cases is not included in the final analysis.

*(also bolded) Statistically significant at *p* < 0.05.

In boys, emotional eating at age 14 was predicted by AN PGS [*ß* = 0.2583 (0.1096), *p* = 0.019] as well as AN/OCD PGS [*ß* = 0.2371 (0.1109), *p* = 0.033] (Table 3). None of the eating disorder phenotypes were predicted by OCD PGS in boys.

**Table 3.** Prediction of eating disorder, obsessive-compulsive disorder, and anxiety symptom dimensions and diagnoses using polygenic scores in boys^a^

Phenotype	Sample size	PGS	*ß*	s.e.	Test statistic^b^	*p*
*Eating disorder symptom dimensions and diagnoses*						
Body image distortion at age 10	2246	AN	0.0059	0.0136	0.430	0.667
		OCD	−0.0260	0.0137	−1.898	0.058
		AN/OCD	0.0103	0.0138	0.746	0.456
Fear of weight gain at age 14	1850	AN	0.0073	0.0113	0.643	0.520
		OCD	−0.0010	0.0114	−0.090	0.928
		AN/OCD	0.0123	0.0115	1.073	0.283
Pressure to lose weight at age 14	1853	AN	0.0490	0.0324	1.512	0.131
		OCD	−0.0095	0.0327	−0.290	0.772
		AN/OCD	0.0425	0.0328	1.296	0.195
Restraint at age 14	1817	AN	0.0221	0.0206	1.070	0.285
		OCD	−0.0054	0.0208	−0.260	0.795
		AN/OCD	0.0193	0.0209	0.923	0.356
Emotional eating at age 14	1760	AN	0.2583	0.1096	2.357	0.019*
		OCD	−0.0289	0.1110	−0.260	0.795
		AN/OCD	0.2371	0.1109	2.139	0.033*
External eating at age 14	1540	AN	0.0720	0.0866	0.832	0.406
		OCD	0.1174	0.0885	1.327	0.185
		AN/OCD	0.0562	0.0871	0.645	0.519
Thin ideal internalization at age 14	1770	AN	0.0792	0.0707	1.121	0.262
		OCD	0.0002	0.0708	0.002	0.998
		AN/OCD	0.0847	0.0715	1.184	0.237
Body dissatisfaction at age 14	1872	AN	0.2237	0.1620	1.381	0.168
		OCD	−0.0538	0.1633	−0.330	0.742
		AN/OCD	0.2364	0.1637	1.444	0.149
Weight and shape concern at age 14	1865	AN	0.0366	0.0338	1.083	0.279
		OCD	−0.0323	0.0340	−0.951	0.342
		AN/OCD	0.0305	0.0342	0.892	0.372
AN at age 14^c^^,^^d^	28 cases, 1887 controls	AN	–	–	–	–
		OCD	–	–	–	–
		AN/OCD	–	–	–	–
Bulimia nervosa or subthreshold bulimia nervosa at age 14^c^^,^^d^	19 cases, 1896 controls	AN	–	–	–	–
		OCD	–	–	–	–
		AN/OCD	–	–	–	–
Binge-eating disorder or subthreshold binge-eating disorder at age 14^c^^,^^d^	8 cases, 1907 controls	AN	–	–	–	–
		OCD	–	–	–	–
		AN/OCD	–	–	–	–
Eating disorders not otherwise specified or purging disorder at age 14^c^	174 cases, 1741 controls	AN	0.1147	0.0808	1.421	0.155
		OCD	0.0455	0.0805	0.566	0.572
		AN/OCD	0.1395	0.0817	1.707	0.088
Any threshold/subthreshold eating disorder at age 14^c^	229 cases, 1686 controls	AN	0.1107	0.0715	1.548	0.122
		OCD	−0.0200	0.0712	−0.279	0.780
		AN/OCD	0.1151	0.0723	1.591	0.112
Fasting at age 14	1858	AN	−0.0009	0.0036	−0.243	0.808
		OCD	0.0020	0.0036	0.561	0.575
		AN/OCD	0.0007	0.0036	0.183	0.855
Purging at age 14	1854	AN	−0.0030	0.0034	−0.878	0.380
		OCD	−0.0011	0.0034	−0.326	0.744
		AN/OCD	−0.0007	0.0035	−0.188	0.851
Binge eating at age 14	1878	AN	0.0080	0.0083	0.963	0.336
		OCD	0.0011	0.0083	0.133	0.894
		AN/OCD	0.0099	0.0084	1.185	0.236
Compulsive exercise at age 14	1787	AN	0.0155	0.0132	1.176	0.240
		OCD	0.0164	0.0133	1.230	0.219
		AN/OCD	0.0168	0.0134	1.257	0.209
AN at age 16^c^	15 cases, 1476 controls	AN	–	–	–	–
		OCD	–	–	–	–
		AN/OCD	–	–	–	–
Bulimia nervosa at age 16^c^	25 cases, 1466 controls	AN	–	–	–	–
		OCD	–	–	–	–
		AN/OCD	–	–	–	–
Binge-eating disorder at age 16^c^	10 cases, 1481 controls	AN	–	–	–	–
		OCD	–	–	–	–
		AN/OCD	–	–	–	–
Eating disorders not otherwise specified or purging disorder at age 16^c^	236 cases, 1255 controls	AN	0.0317	0.0712	0.445	0.656
		OCD	0.0926	0.0719	1.287	0.198
		AN/OCD	0.0121	0.0721	0.167	0.867
Any threshold/subthreshold eating disorder at age 16^c^	286 cases, 1205 controls	AN	0.0402	0.0660	0.609	0.543
		OCD	0.0618	0.0667	0.928	0.354
		AN/OCD	0.0338	0.0669	0.504	0.614
Fasting at age 16	1407	AN	0.0010	0.0084	0.114	0.909
		OCD	−0.0010	0.0084	−0.113	0.910
		AN/OCD	−0.0021	0.0085	−0.248	0.804
Purging at age 16	1412	AN	0.0024	0.0047	0.505	0.614
		OCD	0.0073	0.0046	1.582	0.114
		AN/OCD	0.0025	0.0047	0.529	0.597
Binge eating at age 16	1214	AN	0.0132	0.0128	1.033	0.302
		OCD	−0.0171	0.0129	−1.325	0.185
		AN/OCD	0.0223	0.0129	1.723	0.085
Compulsive exercise at age 16	1346	AN	0.0169	0.0197	0.860	0.390
		OCD	0.0152	0.0195	0.782	0.435
		AN/OCD	0.0124	0.0199	0.620	0.535
*Obsessive-compulsive disorder symptom dimensions and diagnosis*						
OCD at age 10^c^^,^^d^	15 cases, 2610 controls	AN	–	–	–	–
		OCD	–	–	–	–
		AN/OCD	–	–	–	–
OCD latent factor – symmetry, checking at age 10	2607	AN	0.0187	0.0104	1.794	0.073
		OCD	−0.0073	0.0105	−0.699	0.484
		AN/OCD	0.0193	0.0105	1.830	0.067
OCD latent factor – dirt/germs at age 10	2607	AN	0.0157	0.0087	1.798	0.072
		OCD	−0.0014	0.0088	−0.155	0.877
		AN/OCD	0.0160	0.0089	1.804	0.071
OCD at age 13^c^^,^^d^	6 cases, 2419 controls	AN	–	–	–	–
		OCD	–	–	–	–
		AN/OCD	–	–	–	–
OCD latent factor – symmetry, checking at age 13	2411	AN	0.0139	0.0144	0.961	0.337
		OCD	−0.0183	0.0145	−1.262	0.207
		AN/OCD	0.0154	0.0145	1.062	0.288
OCD latent factor – dirt/germs at age 13	2411	AN	−0.0297	0.0132	−2.243	0.025*
		OCD	−0.0177	0.0133	−1.327	0.185
		AN/OCD	−0.0300	0.0133	−2.246	0.025*
*Anxiety symptom dimensions and diagnoses*						
Separation anxiety at age 7^c^	80 cases, 2670 controls	AN	0.0521	0.1152	0.452	0.651
		OCD	−0.0697	0.1127	−0.619	0.536
		AN/OCD	0.0571	0.1163	0.491	0.624
Specific phobia at age 7^c^	51 cases, 2701 controls	AN	0.1224	0.1430	0.856	0.392
		OCD	−0.0699	0.1407	−0.497	0.619
		AN/OCD	0.1510	0.1444	1.046	0.296
Latent factor – physical anxiety at age 10	2607	AN	0.0181	0.0143	1.264	0.206
		OCD	−0.0147	0.0145	−1.020	0.308
		AN/OCD	0.0111	0.0145	0.763	0.445
Latent factor – worrying at age 10	2607	AN	0.0292	0.0133	2.197	0.028*
		OCD	0.0057	0.0134	0.424	0.671
		AN/OCD	0.0171	0.0134	1.274	0.203
Latent factor – social phobia at age 10	2607	AN	0.0117	0.0124	0.947	0.344
		OCD	−0.0254	0.0125	−2.037	0.042*
		AN/OCD	0.0061	0.0125	0.486	0.627
Social phobia at age 13^c^	25 cases, 2399 controls	AN	–	–	–	–
		OCD	–	–	–	–
		AN/OCD	–	–	–	–
Generalized anxiety disorder at age 13^c^	54 cases, 2370 controls	AN	0.1184	0.1376	0.861	0.390
		OCD	0.1169	0.1389	0.841	0.400
		AN/OCD	0.1658	0.1386	1.196	0.232
Latent factor – physical anxiety at age 13	2411	AN	−0.0118	0.0140	−0.838	0.402
		OCD	−0.0052	0.0141	−0.371	0.710
		AN/OCD	−0.0091	0.0141	−0.643	0.520
Latent factor – worrying at age 13	2411	AN	0.0012	0.0153	0.077	0.939
		OCD	0.0017	0.0154	0.109	0.914
		AN/OCD	−0.0022	0.0154	−0.143	0.886
Latent factor – social phobia at age 13	2411	AN	0.0200	0.0147	1.364	0.173
		OCD	−0.0023	0.0148	−0.154	0.877
		AN/OCD	0.0078	0.0148	0.527	0.598
Generalized anxiety disorder at age 15^c^	42 cases, 1813 controls	AN	–	–	–	–
		OCD	–	–	–	–
		AN/OCD	–	–	–	–

Abbreviations: PGS, polygenic score; *ß*, standardized beta regression coefficient; s.e., standard error; AN, anorexia nervosa; OCD, obsessive-compulsive disorder; AN/OCD, anorexia nervosa/obsessive-compulsive transdiagnostic phenotype.

a Genomic principal components 1–5 were used as covariates to account for population stratification.

b We report *t*-values for continuous phenotypes and *z*-values for binary phenotypes.

c Binary phenotype.

d Due to insufficient statistical power, any binary measure with less than 50 cases is not included in the final analysis.

*(also bolded) Statistically significant at *p* < 0.05.

### OCD and anxiety symptom phenotypes and diagnoses

In girls, AN PGS predicted a higher score for OCD latent factor dirt/germs at age 13 [*ß* = 0.0281 (0.0129), *p* = 0.030] and an increased likelihood of separation anxiety at age 7 [*ß* = 0.4342 (0.1246), *p* = 0.001] (Table 2). AN/OCD PGS predicted an increased likelihood of separation anxiety [*ß* = 0.4868 (0.1246), *p* < 0.001] as well as higher scores for latent factors OCD dirt/germs [*ß* = 0.0277 (0.0128), *p* = 0.031], worrying [*ß* = 0.0334 (0.0164), *p* = 0.042], and social phobia at age 13 [*ß* = 0.0367 (0.0155), *p* = 0.018]. OCD PGS did not predict any of the OCD or anxiety phenotypes.

In boys, AN PGS predicted a higher score for latent factor worrying at age 10 [*ß* = 0.0292 (0.0133), *p* = 0.028] but a lower score for OCD latent factor dirt/germs at age 13 [*ß* = −0.0297 (0.0132), *p* = 0.025] (Table 3). AN/OCD PGS also negatively predicted OCD latent factor dirt/germs at age 13 [*ß* = −0.0300 (0.0133), *p* = 0.025], whereas OCD PGS predicted a lower score for latent factor social phobia at age 10 [*ß* = −0.0254 (0.0125), *p* = 0.042].

## Discussion

In this exploratory study, we were able to predict eating disorder, OCD, and anxiety phenotypes using AN, OCD, and AN/OCD PGS in girls and boys separately during different developmental points in a large population sample. The majority of phenotypes predicted by AN PGS were also predicted by AN/OCD PGS (e.g. emotional eating at age 14 in boys; separating anxiety at age 7 in girls). However, this overlap was not 100% (e.g. latent factor worrying at age 10 in boys predicted by AN and not AN/OCD PGS), and none of the phenotypes predicted by OCD PGS were also predicted by AN/OCD PGS, suggesting that the genetic risk associated with some phenotypes may be more OCD-specific than being based on a transdiagnostic common factor. Notably, there were no phenotypes predicted separately by both AN and OCD PGS. Considering the notably smaller sample size of the OCD GWAS compared to the AN GWAS (2688 *v.* 16 992 cases), OCD PGS is likely to be underpowered, and some phenotypes associated with a higher genetic load for OCD may be predicted by the AN/OCD PGS. There were also phenotypes only predicted by the transdiagnostic PGS (e.g. thin ideal internalization at age 14 in girls), further demonstrating the likely boost in statistical power for both AN and OCD with the use of the transdiagnostic genotype.

Compulsive exercise was the only intermediate phenotype that was positively predicted by more than one disorder-specific PGS in girls, suggesting it may be a key intermediate phenotype that, although commonly associated with eating disorders, is influenced by genetic risk for both AN and OCD. Together with evidence for shared genetic risk between a broad AN phenotype and general propensity for physical activity (Watson et al., 2019), this finding suggests that genetic factors may be particularly relevant to understanding the development of compulsive exercise in eating disorders. Compulsive exercise encompasses many of the hallmark symptoms of AN (e.g. weight and shape concern) and OCD (e.g. compulsive behavior) (Davis & Kaptein, 2006). Furthermore, comorbid OCD symptoms are especially pronounced in the subpopulation of AN patients with compulsive exercise (Błachno et al., 2016; Davis & Claridge, 1998; Davis & Kaptein, 2006; Davis, Katzman, & Kirsh, 1999; Naylor, Mountford, & Brown, 2011), which has significant clinical relevance since the presence of compulsive exercise in AN is an established predictor of treatment outcomes, including higher pathology at discharge from inpatient treatment (Dalle Grave, Calugi, & Marchesini, 2008), relapse (Carter, Blackmore, Sutandar-Pinnock, & Woodside, 2004), and greater energy requirements for weight gain (Kaye, Gwirtsman, Obarzanek, & George, 1988). Treatments for this symptom are currently lacking, and our preliminary results point to the need for additional investigation of the habitual and compulsive nature of exercise behavior in girls, which may lead to targeted intervention development for this symptom that derives from a modern biobehavioral understanding of both eating disorders and OCD. It is not clear why this association was not present in boys, but one potential explanation is the lack of statistical power for current PGS to detect such a relationship in males, which may require much larger discovery sample sizes. Alternatively, the risk associated with compulsive exercise may be driven by mechanisms outside of AN or OCD genetic load in men.

Importantly, our findings suggest the presence of both sex and developmental timing influences in the biological pathways and vulnerabilities leading to these symptom phenotypes. Contrary to our hypothesis, our results show that AN genetics may play a more prominent role in risk for eating disorder and related phenotypes in girls, as compared to boys, and especially in early development. In fact, significant eating disorder symptom phenotypes at age 14 – but none at age 16 – were predicted by AN PGS. Variability in genetic influence depending on the stage of development has previously been established, as twin studies have shown changes in the contribution of genetic and environmental risk factors for disordered eating during different stages of adolescence (Fairweather-Schmidt & Wade, 2015; O'Connor, Culbert, Mayhall, Burt, & Klump, 2020). However, the twin-based heritability estimate for disordered eating has been shown to be much higher in boys than girls prior to puberty (0.52 in boys *v.* 0 in girls) (Klump et al., 2012), suggesting that AN genetic load could manifest itself earlier in boys, which is not what we observed in our study. Except for body image distortion at age 10, all eating disorder phenotypic data were collected at age 14 onward, so we cannot rule out the possibility that AN PGS may predict eating disorder phenotypes in boys at an earlier age than we have data available for. Another possible explanation is that the risk for disordered eating in boys could be attributed to a higher genetic load for other eating disorders – for which currently no large GWAS results exist – or other phenotypes independent of AN. Additionally, PGS is designed to account for common genetic variation, therefore genetic risk for eating disorders in males could be potentially driven by other types of variation such as copy number variants, rare variants, epigenetic factors, or other genetic mechanisms that PGS does a poor job of capturing.

Genetic prediction of anxiety symptoms and diagnoses also showed notable differences in boys and girls. For instance, AN PGS predicted separation anxiety at age 7 in girls and increased worrying at age 10 in boys. Epidemiological studies show over a 10-fold increase in AN risk among girls with separation anxiety disorder (Bulik, Sullivan, Fear, & Joyce, 1997), and a twin-based study reported a shared genetic effect influencing liability to AN, separation anxiety, and childhood overanxious disorder (which is very similar to generalized anxiety disorder in adults) during different stages of development (Silberg & Bulik, 2005), supporting our findings about the presence of a shared genetic pathway between anxiety and AN. We unexpectedly observed that lower OCD-specific genetic risk predicted lower scores on the latent factor indexing social phobia at age 13 in boys. While anxiety symptoms are common in patients with OCD, OCD is distinct from anxiety disorders phenotypes – in fact it is now a separate diagnostic chapter in DSM – and our results suggest that OCD may be distinct from anxiety disorders at a genetic level, especially for men. Replication of these associations is required to better understand the nature of these relationships and the importance of potential sex differences in the biological pathways associated with anxiety risk.

Notably, significant PGS predictions did not always fall cleanly in accordance with hypothesized disorder-specific symptom phenotypes, especially in the case of anxiety phenotypes. For instance, AN – but not OCD – PGS predicted higher scores for OCD-dirt/germs and worrying during different developmental timepoints in girls. While contamination fears are often associated with OCD, they are not unique to OCD and have a cross-disorder component. In fact, it is not uncommon for individuals with AN to present with food-related contamination fears (Drummond & Kolb, 2008). In our previous ALSPAC study, we found that the latent factor worrying significantly predicts eating disorder symptoms at ages 14 and 16 as well as AN diagnosis at age 16 (Schaumberg et al., 2019). This may suggest that uncontrolled worrying may be an underlying early symptom of AN and disordered eating that precedes the manifestation of an eating disorder.

Our study has notable strengths that merit consideration. This is the first study to use AN, OCD, or AN/OCD PGS to predict eating disorder and anxiety intermediate phenotypes in a large population sample through a developmental perspective and also to examine how genetic risk may manifest differently in boys and girls. We augmented the diagnostic approach by including intermediate phenotypes measured continuously to capture the full range of these underlying traits in the general population. Furthermore, studying these associations in the general population allows a finer understanding of intermediate phenotypes and broader psychopathology as treatment-seeking individuals might have notable differences from the general population (e.g. increased comorbid psychopathology).

Limitations of our study include reliance on self- or parent-report symptoms instead of clinical diagnoses, phenotype data being available for only a subset of participants with the potential for response bias, and potential Type 2 error due to lack of statistical power for PGS in the prediction of genetic risk. The effect sizes observed were relatively small, and due to the exploratory nature of our study with the aim of elucidating sex differences using transdiagnostic prediction and symptom-level data, we did not correct for multiple testing with the hopes of generating potential hypotheses for future work. Of note, few participants met diagnostic criteria for AN, bulimia nervosa, or binge-eating disorder, whereas we had better statistical power for the non-specific eating disorder statuses (especially in girls), which likely explains why PGS did not predict AN diagnosis in either sex and none of the eating disorder diagnoses in boys. Similarly, a high probability (50% or higher) for anxiety disorder diagnoses was uncommon despite our attempt to increase power through dichotomizing these items, and even with dichotomizing, we did not have enough cases to include OCD diagnosis in our outcomes. Additionally, we did not address the presence of comorbid psychiatric diagnoses, therefore we cannot account for the role of comorbidities or the genetic risk associated with these additional diagnoses. However, as comorbidity is the norm and not the exception, our results are likely to capture associations that are more likely to be present in clinical and population settings, as pure forms of eating disorders and OCD are not common. With the exception of body image distortion at age 10, all eating disorder data were collected at age 14 onward, therefore we were unable to examine the potential association between PGS and eating behavior in early childhood. From a genetic perspective, whether all of the eating disorder-related symptom phenotypes examined as a part of our study actually fall on an etiological continuum with AN is not clear (Dinkler et al., 2021). Finally, the AN PGS was constructed using a much larger GWAS than the OCD GWAS, which may have translated to OCD PGS being underpowered and the AN/OCD transdiagnostic GWAS being more heavily skewed by AN PGS than OCD PGS.

Taken together, results of our study provide preliminary support for utilizing the high positive genetic correlation between AN and OCD (Watson et al., 2019), leading to a small boost in predictive power through the use of a transdiagnostic PGS. We anticipate this statistical boost to become more notable as AN and (especially) OCD GWAS sample sizes continue to increase. Furthermore, our findings also point to differences in the manifestation of genetic risk for eating disorder and anxiety symptoms in boys and girls. Genetic risk associated with AN may be a stronger predictor of eating disorder symptoms earlier in development, whereas OCD genetic risk – albeit limited based on current GWAS data – may increase in effect across adolescence. Another significant observation was that compulsive exercise may be an intermediate phenotype or clinical manifestation of shared genetic risk factors for both AN and OCD. Compulsive exercise might be a distinct AN subphenotype, and clinical research should continue to explore habitual and compulsive processes associated with this symptom. Finally, this study opens up new avenues for a clearer understanding of biology of behaviors and intermediate phenotypes in eating disorders.

## Supplementary material

For supplementary material accompanying this paper visit https://doi.org/10.1017/S0033291721005079. click here to view supplementary material
