# Supplementary material for: Predicting eating disorder and anxiety symptoms using disorder-specific and transdiagnostic polygenic scores for anorexia nervosa and obsessive-compulsive disorder
Source: Psychol Med. 2022 Mar 4;53(7):3021–35. doi: 10.1017/S0033291721005079 (PMC9440960; doi:10.1017/S0033291721005079)
Supplement: Supplementary file 1 [file S0033291721005079sup001.docx]

**Supplementary Information**

**TABLE OF CONTENTS**

| **Supplementary Text** | **2** |
| --- | --- |
| Assessments | 2 |
| Anorexia Nervosa/Obsessive-Compulsive Disorder Transdiagnostic Genome-Wide Association Meta-Analysis | 5 |
| Polygenic Score Calculation | 8 |
| References | 9 |
| **Supplementary Tables** | **14** |
| Table S1 | 14 |
| Table S2 | 15 |
| Table S3 | 16 |
| Table S4 | 21 |
| **Supplementary Figures** | **26** |
| Figure S1 | 26 |
| **Consortia Co-Authors** | **27** |
| Anorexia Nervosa Genetics Initiative | 27 |
| Eating Disorders Working Group of the PGC | 27 |
| Obsessive Compulsive Disorder Working Group of the PGC | 29 |

**Assessments**

**Youth Risk Behavior Surveillance System Questionnaire**

For each ED behavior, questions inquired about the previous year and were adapted from the Youth Risk Behavior Surveillance System (YRBSS) questionnaire (Kann et al., 1996), which were validated in an epidemiological study of youth (Field, Taylor, Celio, & Colditz, 2004). Adolescents self-reported eating disorder behaviors at ages 14 and 16. Binge eating was defined as eating a very large amount of food and feeling out of control during these episodes. Purging was defined as making oneself sick or using laxatives to lose weight or avoid gaining weight. Fasting was assessed with the question “During the past year, how often did you fast (not eat for at least half a day) to lose weight or avoid gaining weight?”. Participants rated their frequency of binge eating, purging, and fasting, on a Likert scale of 0 - never, 1- less than once a month, 2 – one to three times a month, or 4 – once a week or more, which were treated as continuous variables in the current analyses. Presence of compulsive exercise was defined as reporting exercise for weight loss or to avoid weight gain, associated with: (1) difficulty with chores/schoolwork due to the time spent exercising; (2) exercising even when sick or injured, or (3) feeling guilty about missing an exercise session. At age 14, participants rated their frequency of engaging in exercise to lose weight. At age 16, exercise was assessed by a question asking whether participants exercised for weight loss and experienced guilt due to missing an exercise session.

**Development And Wellbeing Assessment**

Parentally-reported obsessive-compulsive disorder (OCD) and anxiety symptoms at age 7, 10, and 13, and adolescent-reported OCD and anxiety symptoms at age 15 were collected using the Development And Wellbeing Assessment (DAWBA) (A. Goodman, Heiervang, Collishaw, & Goodman, 2011; R. Goodman, Ford, Richards, Gatward, & Meltzer, 2000), a semi-structured interview designed to assess psychopathology in children ages 4-16. Parent-report was utilized for younger ages as study comparing self- and parent-report versions of the DAWBA favored a parent-report format for identifying cases and discriminating clinical and community samples (Kuhn et al., 2017). The DAWBA comprises 83 symptom-based items reflecting diagnostic criteria, and diagnoses derived from these items have good agreement with clinical case note diagnoses (R. Goodman et al., 2000). Probabilities of anxiety disorder diagnoses at ages 7 (specific phobia and separation anxiety), 10 (OCD), 13 (OCD, social phobia, and generalized anxiety disorder), and 15 (generalized anxiety disorder) were determined using computer‐generated DAWBA band variables (A. Goodman et al., 2011). Briefly, DAWBA scoring assigns the probability of the participant meeting DSM-IV criteria for an anxiety disorder (i.e., 0 ≤ 0.1% likelihood; 1 = 0.5% likelihood; 2 = 3% likelihood; 3 = 15% likelihood; 4 = 50% likelihood; 5 ≥ 70% likelihood). However, due to a very small number of participants scoring high on these DAWBA diagnostic variables, we dichotomized these items to capture whether the likelihood of a diagnosis was less than 50% versus 50% or higher (i.e., scores 0­–3 recoded as “absent” and scores 4–5 recoded as “present”).

**Eating Disorder Cognitions**

Body image distortion at age 10 was assessed using the Stunkard Figure Rating Scale, which assesses discrepancy between a child’s actual and ideal body size (Stunkard, Sorensen, & Schulsinger, 1983). A slightly modified version of the Dutch Eating Behavior Questionnaire (DEBQ) was administered at age 14 (Stice, Mazotti, Krebs, & Martin, 1998; Van Strien, Frijters, Bergers, & Defares, 1986). The DEBQ raw scores are totaled into three subscale scores: Emotional Eating (eating in reaction to emotions - boys Cronbach *α*=0.89; girls Cronbach *α*=0.90), External Eating (eating in reaction to external cues boys Cronbach *α*=0.68; girls Cronbach *α*=0.66), and Restrained Eating (cognitively attempting to limit one’s caloric intake). All emotional and external eating items were used in this study; only two items of the restraint subscale were used as a measure of restraint. Higher scores on each subscale indicate greater symptomatology.

Body dissatisfaction was assessed using the Body Dissatisfaction Scale part of the Satisfaction and Dissatisfaction With Body Parts Scale (boys Cronbach α=0.89; girls Cronbach *α*=0.90 (Berscheid, Walster, & Bohrnstedt, 1973)); thin-ideal internalization was assessed using the Ideal-Body Stereotype Scale-Revised (Stice et al., 1998; Stice, Ziemba, Margolis, & Flick, 1996); with gender-specific questions; girls were asked five questions (Cronbach *α*=0.56) and boys six questions (Cronbach *α*=0.71 (Calzo, Austin, & Micali, 2018)); whereas pressure to lose weight was assessed using four items adapted from the Perceived Sociocultural Pressure Scale (boys Cronbach *α*=0.56; girls Cronbach α=0.56 (Stice et al., 1996)). Weight and shape concern was captured at ages 14 and 16 using three questions from the McKnight Risk Factor Survey (Micali et al., 2015; Shisslak et al., 1999).

**Obsessive-Compulsive Disorder and Anxiety Latent Factors**

DAWBA latent OCD and anxiety factors (obsessive compulsive disorder [OCD] latent factor symmetry; OCD latent factor dirt/germs; physical anxiety; worrying; social phobia) were defined at ages 10 and 13 (Schaumberg et al., 2019). OCD and anxiety factor loadings from age 10 were previously defined (Schaumberg et al., 2019), and these factors were utilized in the current study. In replication of methods used to derive age 10 factors, screened items were included in an exploratory factor analysis (EFA) with a Geomin rotation (oblique) to identify latent dimensions of anxiety symptoms at age 13. Prior to analyses, appropriateness of items was identified for the factor analysis. Items were excluded that were: (1) related to eating; (2) primarily focused on the duration or frequency of a symptom (e.g., symptom is present for at least two weeks); (3) assessed functional interference secondary to symptoms (e.g., these acts or thoughts have interfered with family relationships); or (4) qualified a symptom for diagnostic criteria (e.g., child recognizes fears as excessive). Items that fit poorly on all factors were also identified and removed from analyses until a final factor structure was identified at age 13.

**Anorexia Nervosa/Obsessive-Compulsive Disorder Transdiagnostic Genome-Wide Association Meta-Analysis**

**Methods**

The genetic data for the anorexia nervosa (AN) and OCD transdiagnostic meta-analysis came from the summary statistics of the largest and most recently published genome-wide association study (GWAS) for AN (Watson et al., 2019) and OCD (International Obsessive Compulsive Disorder Foundation Genetics Collaborative (IOCDF-GC) and OCD Collaborative Genetics Association Studies (OCGAS), 2018). For AN, we used data from the Eating Disorders Working Group of the Psychiatric Genomics Consortium (PGC-ED) Freeze 2 GWAS of 16,992 cases and 55,525 controls. For OCD, we used data derived from the most recent published PGC-OCD Freeze 1 GWAS of 2,688 cases and 7,031 controls. All participants were of European ancestry. Using these data sets, we conducted a meta-analysis of 19,680 AN/OCD cases and 62,556 controls. Single nucleotide polymorphism (SNP)-based relatedness testing did not show sample overlap between the AN and OCD studies (*pi-hat* > 0.1 from linkage disequilibrium (LD)-pruned sets of SNPs in PLINK v1.9 (Chang et al., 2015; Purcell et al., 2007).

We followed the same methodology as our previously published AN/OCD cross-disorder GWAS (Yilmaz et al., 2020) which used the same OCD dataset but an older and smaller AN GWAS (Duncan et al., 2017). As a part of the pre-processing step, we identified and only included SNPs that met the following criteria for each dataset: (1) minor allele frequency (MAF) > 0.01; (2) imputation quality (INFO) score > 0.6; (3) ≥ 60% of cases and controls from the overall meta-analysis per trait available for each variant; and (4) not an A/T or G/C variant with frequencies between 0.4 and 0.6. We parsed both input summary statistic files from AN and OCD to identify variants that met these criteria and found 7,913,856 passing variants in the AN data and 7,707,306 passing variants in the OCD data. We aligned variants between the datasets based on the chromosome and position they mapped to alongside the listed alleles 1 and 2. We found that between the AN and OCD datasets, 3,040,189 were exact matches and 3,537,060 could be matched via an allele flip in one summary statistics file. Additional strand flipping did not result in any additional matching. This made for a total of 6,577,249 variants present in both datasets that could be properly matched and jointly analyzed. The meta-analysis was conducted using the PGC’s Ricopili pipeline (see <https://sites.google.com/a/broadinstitute.org/ricopili>). This pipeline utilizes METAL (Willer, Li, & Abecasis, 2010) and takes into account sample sizes as well as strength in direction of effect in each separate data set (inverse-variance weighted meta-analysis using a fixed-effects model). The meta-analysis summary statistics files for AN and OCD used in the transdiagnostic analysis are publicly available for download on the PGC website (<http://www.med.unc.edu/pgc/results-and-downloads>).

**Results**

As shown in the quantile–quantile (QQ) plot (Supplementary Figure S1a), the genomic inflation factor (*λ_1000_*) of 1.007 indicates no signs of excessive inflation. Inflation statistics from individual AN (Watson et al., 2019) and OCD (International Obsessive Compulsive Disorder Foundation Genetics Collaborative (IOCDF-GC) and OCD Collaborative Genetics Association Studies (OCGAS), 2018) data sets appear similarly well controlled (λ_1000_ values of 1.010 and 1.009, respectively).

In the transdiagnostic GWAS, seven variants reached genome-wide significance at *P* < 5 × 10^−8^ (Supplementary Figure S1b and Supplementary Table S1). Loci #1 (chr3:47588390­–51368390; lead SNP: rs9877501; *P* = 2.225 × 10^-14^), #2 (chr11:114997229–115363429; lead SNP: rs17649730; *P* = 7.075 × 10^-10^), #3 (chr1:96699119–97284119; lead SNP: rs2181375, *P* = 1.311 × 10^-8^), and #4 (chr2:53874323–54363223, lead SNP: rs12613353; *P* = 1.606 × 10^-8^) were also genome-wide significant in the original AN GWAS (Watson et al., 2019), whereas the original OCD GWAS did not yield any genome-wide significant hits (International Obsessive Compulsive Disorder Foundation Genetics Collaborative (IOCDF-GC) and OCD Collaborative Genetics Association Studies (OCGAS), 2018). In addition to these overlapping loci with AN, three of our significant regions were novel. Locus #5 (chr 10: 75778228–76496228; lead SNP: rs7916143; *P* = 4.287 × 10^-8^) spans over four genes, and the nearest *ADK* gene is important in the regulating concentrations of both extracellular adenosine and intracellular adenine nucleotides. Locus #6 (chr1:193010527–19340252, lead SNP: rs10754051, *P* = 4.426 × 10^-8^) spans over six genes. The nearest genes are *CDC73* (a tumor suppressor), *MIR1278* (involved in the stability and translation of mRNAs), and *B3GALT2* (encodes type II membrane-bound glycoproteins). Finally, locus #7 (chr1:47588390–51368390; lead SNP: rs2821359; *P* = 4.662 × 10^-8^) is a single gene locus that harbors NR5A2, which is important regulator of embryonic development.

Of note, the prominent but non-significant signal we detected near the MHC (index SNP rs75063949, chr6:25591041) in our previous cross-disorder meta-analysis (Yilmaz et al., 2020) was no longer in the suggestive zone (new *P* = 0.1757).

**Polygenic Score Calculation**

Polygenic scores (PGS) for AN, OCD, and AN/OCD were calculated using PRS-CS, which infers posterior SNP effect sizes under continuous shrinkage priors using GWAS summary statistics and an external LD reference panel (Ge, Chen, Ni, Feng, & Smoller, 2019). For our target dataset, we started with post-QC and post-imputation genotypes of 7,977 individuals in the Avon Longitudinal Study of Parents and Children (ALSPAC) cohort (more information on genotyping and imputation processes of ALSPAC genetic data is available elsewhere (Martin, Hamshere, Stergiakouli, O'Donovan, & Thapar, 2014; Paternoster et al., 2012)). Upon obtaining data, we first performed additional QC to ensure high-quality data analysis using the following parameters in PLINK v1.9: (1) drop duplicate SNPs and variants without valid rsid’s; (2) keep SNPs with INFO ≥ 0.8; (3) remove SNPs with missingness > 0.02; (4) keep SNPs with MAF ≥ 0.05; (5) remove subjects with missingness > 0.02; and (6) remove one twin randomly from 13 twin-pairs. A total number of 7,779 individuals (3,992 male and 3,787 female) and 4,248,592 SNPs were in the final QC’d dataset. We then calculated 20 genomic principal components (PCs) for both sexes combined. For PGS calculation, 1000 Genomes European dataset (1000 Genomes Project Consortium et al., 2012) was used as the external LD reference panel. After obtaining posterior SNP effect sizes for AN, OCD, and AN/OCD separately in PRS-CS using the GWAS summary statistics files, we used the *--score* command in PLINK to calculate AN, OCD, and AN/OCD PGS in the ALSPAC target sample. Using R (R Core Team, 2017) v4.0.2, we calculated z-scores for each of the three PGS in ALSPAC after removing participants with no phenotype data, which brought our combined target sample size to 6,567 participants (3,270 girls and 3,297 boys). We then performed linear regression for continuous target phenotypes (*glm*) and logistic regression (*glm*; *family="binomial" (link='logit')*) for binary target phenotypes for the prediction of each of the 27 eating disorder, 6 OCD, and 11 anxiety intermediate phenotypes and diagnoses in the ALSPAC sample by AN, OCD, and AN/OCD PGS separately. Regression models included the first five genomic PCs as covariates in all analyses to account for population stratification. We ran the analyses in the combined sample both with sex included as a covariate as well as without sex as a covariate. All genetic analyses were performed using the SURFsara Lisa computing cluster (<https://userinfo.surfsara.nl/systems/lisa>).

**References**

1000 Genomes Project Consortium, Abecasis, G. R., Auton, A., Brooks, L. D., DePristo, M. A., Durbin, R. M., . . . McVean, G. A. (2012). An integrated map of genetic variation from 1,092 human genomes. *Nature, 491*(7422), 56-65. doi:10.1038/nature11632

Berscheid, E., Walster, E., & Bohrnstedt, G. (1973). The happy American body: A survey report. *Psychology Today, 7*, 119–131.

Calzo, J., Austin, S., & Micali, N. (2018). Sexual orientation disparities in eating disorder symptoms among adolescent boys and girls in the UK. *European Child and Adolescent Psychiatry*. doi:10.1007/s00787-018-1145-9

Chang, C. C., Chow, C. C., Tellier, L. C., Vattikuti, S., Purcell, S. M., & Lee, J. J. (2015). Second-generation PLINK: rising to the challenge of larger and richer datasets. *Gigascience, 4*, 7. doi:10.1186/s13742-015-0047-8

Duncan, L., Yilmaz, Z., Gaspar, H., Walters, R., Goldstein, J., Anttila, V., . . . Bulik, C. M. (2017). Significant locus and metabolic genetic correlations revealed in genome-wide association study of anorexia nervosa. *American Journal of Psychiatry, 174*(9), 850-858. doi:10.1176/appi.ajp.2017.16121402

Field, A. E., Taylor, C. B., Celio, A., & Colditz, G. A. (2004). Comparison of self-report to interview assessment of bulimic behaviors among preadolescent and adolescent girls and boys. *International Journal of Eating Disorders, 35*(1), 86-92. doi:10.1002/eat.10220

Ge, T., Chen, C. Y., Ni, Y., Feng, Y. A., & Smoller, J. W. (2019). Polygenic prediction via Bayesian regression and continuous shrinkage priors. *Nature Communications, 10*(1), 1776. doi:10.1038/s41467-019-09718-5

Goodman, A., Heiervang, E., Collishaw, S., & Goodman, R. (2011). The 'DAWBA bands' as an ordered-categorical measure of child mental health: description and validation in British and Norwegian samples. *Social Psychiatry and Psychiatric Epidemiology, 46*(6), 521-532. doi:10.1007/s00127-010-0219-x

Goodman, R., Ford, T., Richards, H., Gatward, R., & Meltzer, H. (2000). The Development and Well-Being Assessment: description and initial validation of an integrated assessment of child and adolescent psychopathology. *Journal of Child Psychology and Psychiatry, 41*(5), 645-655.

International Obsessive Compulsive Disorder Foundation Genetics Collaborative (IOCDF-GC) and OCD Collaborative Genetics Association Studies (OCGAS). (2018). Revealing the complex genetic architecture of obsessive-compulsive disorder using meta-analysis. *Molecular Psychiatry, 23*(5), 1181-1188. doi:10.1038/mp.2017.154

Kann, L., Warren, C., Harris, W., Collins, J., Williams, B., Ross, J., & Kolbe, L. (1996). Youth risk behavior surveillance--United States, 1995. *Journal of School Health, 66*(10), 365-377. Retrieved from <https://www.ncbi.nlm.nih.gov/entrez/query.fcgi?cmd=Retrieve&db=PubMed&dopt=Citation&list_uids=8981266>

Kuhn, C., Aebi, M., Jakobsen, H., Banaschewski, T., Poustka, L., Grimmer, Y., . . . Steinhausen, H. C. (2017). Effective Mental Health Screening in Adolescents: Should We Collect Data from Youth, Parents or Both? *Child Psychiatry and Human Development, 48*(3), 385-392. doi:10.1007/s10578-016-0665-0

Martin, J., Hamshere, M. L., Stergiakouli, E., O'Donovan, M. C., & Thapar, A. (2014). Genetic risk for attention-deficit/hyperactivity disorder contributes to neurodevelopmental traits in the general population. *Biological Psychiatry, 76*(8), 664-671. doi:10.1016/j.biopsych.2014.02.013

Micali, N., De Stavola, B., Ploubidis, G., Simonoff, E., Treasure, J., & Field, A. E. (2015). Adolescent eating disorder behaviours and cognitions: gender-specific effects of child, maternal and family risk factors. *British Journal of Psychiatry, 207*(4), 320-327. doi:10.1192/bjp.bp.114.152371

Paternoster, L., Zhurov, A. I., Toma, A. M., Kemp, J. P., St Pourcain, B., Timpson, N. J., . . . Evans, D. M. (2012). Genome-wide association study of three-dimensional facial morphology identifies a variant in PAX3 associated with nasion position. *American Journal of Human Genetics, 90*(3), 478-485. doi:10.1016/j.ajhg.2011.12.021

Purcell, S., Neale, B., Todd-Brown, K., Thomas, L., Ferreira, M. A., Bender, D., . . . Sham, P. C. (2007). PLINK: a tool set for whole-genome association and population-based linkage analyses. *American Journal of Human Genetics, 81*(3), 559-575. doi:10.1086/519795

R Core Team. (2017). R: A language and environment for statistical computing. Vienna, Austria: R Foundation for Statistical Computing. Retrieved from <https://www.R-project.org/>

Schaumberg, K., Zerwas, S., Goodman, E., Yilmaz, Z., Bulik, C. M., & Micali, N. (2019). Anxiety disorder symptoms at age 10 predict eating disorder symptoms and diagnoses in adolescence. *Journal of Child Psychology and Psychiatry, 60*(6), 686-696. doi:10.1111/jcpp.12984

Shisslak, C. M., Renger, R., Sharpe, T., Crago, M., McKnight, K. M., Gray, N., . . . Taylor, C. B. (1999). Development and evaluation of the McKnight Risk Factor Survey for assessing potential risk and protective factors for disordered eating in preadolescent and adolescent girls. *International Journal of Eating Disorders, 25*(2), 195-214.

Stice, E., Mazotti, L., Krebs, M., & Martin, S. (1998). Predictors of adolescent dieting behaviors: A longitudinal study. *Psychology of Addictive Behaviors, 12*, 195-205.

Stice, E., Ziemba, C., Margolis, J., & Flick, P. (1996). The dual pathway model differentiates bulimics, subclinical bulimics, and controls: Testing the continuity hypothesis. *Behavior Therapy, 27*, 531-549.

Stunkard, A. J., Sorensen, T., & Schulsinger, F. (1983). Use of the Danish Adoption Register for the study of obesity and thinness. *Research Publications - Association for Research in Nervous and Mental Disease, 60*, 115-120.

Van Strien, T., Frijters, J. E. R., Bergers, G., & Defares, P. B. (1986). The Dutch Eating Behavior Questionnaire (DEBQ) for assessment of restrained, emotional, and external eating behavior. *International Journal of Eating Disorders, 5*, 295-315.

Watson, H. J., Yilmaz, Z., Thornton, L. M., Hubel, C., Coleman, J. R. I., Gaspar, H. A., . . . Bulik, C. M. (2019). Genome-wide association study identifies eight risk loci and implicates metabo-psychiatric origins for anorexia nervosa. *Nature Genetics, 51*(8), 1207-1214. doi:10.1038/s41588-019-0439-2

Willer, C. J., Li, Y., & Abecasis, G. R. (2010). METAL: fast and efficient meta-analysis of genomewide association scans. *Bioinformatics (Oxford, England), 26*(17), 2190-2191. doi:10.1093/bioinformatics/btq340

Yilmaz, Z., Halvorsen, M., Bryois, J., Yu, D., Thornton, L. M., Zerwas, S., . . . Crowley, J. J. (2020). Examination of the shared genetic basis of anorexia nervosa and obsessive-compulsive disorder. *Molecular Psychiatry, 25*(9), 2036-2046. doi:10.1038/s41380-018-0115-4

**Table S1. Demographics for the broad sample with a least one eating disorder or anxiety measure between ages 7-16 years and passed genotype quality control**

|  | **Boys (*N*=3,265)** | **Girls (*N*=3,219)** |
| --- | --- | --- |
| Maternal reported eating disorder (at pregnancy or child age 7) | 4.7% | 4.4% |
| Parental social class | 13.6% manual labor | 17.0% manual labor |
| Maternal education | 56.3% GSCE level  43.7% A-level and above | 56.0% GSCE level  44.0% A-level and above |
| Financial problems reported | 27.9% | 29.3% |
| z-BMI score at age 10 | 0.59 (*SD*=0.99) | 0.52 (*SD*=0.93) |
| Underweight at age 14 | 8.5% | 9.6% |
| Overweight/obese at age 14 | 16.9% | 18.9% |

**Table S2. Genome-wide significant loci for anorexia nervosa/obsessive-compulsive disorder transdiagnostic phenotype**

| **Locus** | **Chr** | **BP Region** | | **Lead SNP** | ***P*** | **A1/A2** | ***OR*** | ***SE*** | **Freq** | **Number of Genes** | **Nearest Gene(s)** |
| --- | --- | --- | --- | --- | --- | --- | --- | --- | --- | --- | --- |
|  |  | **Range Left** | **Range Right** |  |  |  |  |  |  |  |  |
| **1** | 3 | 47588390 | 51368390 | rs9877501 | 2.225E-14 | C/G | 0.86649 | 0.0188 | 0.881 | 60 | *SLC26A6, MIR6824, CELSR3, MIR4793, CELSR3-AS1, NCKIPSD, IP6K2* |
| **2** | 11 | 114997229 | 115363429 | rs17649730 | 7.075E-10 | C/T | 0.89404 | 0.0182 | 0.866 | 1 | *CADM1* |
| **3** | 1 | 96699119 | 97284119 | rs2181375 | 1.311E-08 | A/G | 1.07401 | 0.0126 | 0.408 | 1 | *LOC101928241* |
| **4** | 2 | 53874323 | 54363223 | rs12613353 | 1.606E-08 | T/G | 0.90883 | 0.0169 | 0.843 | 7 | *ASB3, GPR75-ASB3, CHAC2, ERLEC1* |
| **5^a^** | 10 | 75778228 | 76496228 | rs7916143 | 4.287E-08 | T/C | 1.0808 | 0.0142 | 0.264 | 4 | *ADK* |
| **6^a^** | 1 | 193010527 | 193402527 | rs10754051 | 4.426E-08 | T/C | 0.93202 | 0.0129 | 0.585 | 6 | *CDC73, MIR1278, B3GALT2* |
| **7^a^** | 1 | 47588390 | 51368390 | rs2821359 | 4.662E-08 | T/C | 0.92146 | 0.015 | 0.781 | 1 | *NR5A2* |

*Note.* Shown are the results of the GWAS meta-analysis of anorexia nervosa (16,992 cases and 55,525 controls) and obsessive-compulsive disorder (2,688 cases and 7,031 controls) which detected seven genome-wide significant loci. Chr (chromosome) and BP (basepair) Region (hg19) are shown for SNPs (single-nucleotide polymorphisms) with *P* < 1E-05 and linkage-disequilibrium (LD) *r*^2^ > 0.1 with the most associated "lead" SNP. A1/A2 refers to Allele 1/Allele 2 and *OR* and *SE* are the odds ratio and standard error for the association between A1 and the phenotype. Freq is the frequency of A1 in controls. Nearest gene(s) is the nearest gene(s) within the region of LD "friends" of the lead variant (LD-*r*^2^ > 0.6 +/- 50 kb). Chromosome X was not included in the analysis. Note that although lead variants are annotated to the nearest gene, this does not mean that the gene listed is a causal gene.

^a^ Novel genome-wide significant loci which were not significant in the anorexia nervosa GWAS (genome-wide association study). Obsessive-compulsive disorder GWAS did not yield any genome-wide significant findings.

**Table S3. Prediction of eating disorder, obsessive-compulsive disorder, and anxiety symptom dimensions and diagnoses using polygenic scores in all participants, with sex as covariate^a^**

| **Eating Disorder Symptom Dimensions and Diagnoses** | | | | | | |
| --- | --- | --- | --- | --- | --- | --- |
| **Phenotype** | **Sample size** | **PGS** | ***ß*** | ***SE*** | **Test statistic^d^** | ***P*** |
| Body image distortion at age 10 | 4758 | AN | 0.001 | 0.010 | 0.090 | 0.928 |
|  |  | OCD | -0.008 | 0.010 | -0.816 | 0.415 |
|  |  | AN/OCD | 0.002 | 0.010 | 0.227 | 0.820 |
| Fear of weight gain at age 14 | 4127 | AN | 0.014 | 0.010 | 1.375 | 0.169 |
|  |  | OCD | 0.005 | 0.010 | 0.524 | 0.601 |
|  |  | AN/OCD | 0.019 | 0.010 | 1.837 | 0.066 |
| Pressure to lose weight at age 14 | 4116 | AN | 0.064 | 0.028 | 2.326 | **0.020^*^** |
|  |  | OCD | 0.006 | 0.027 | 0.220 | 0.826 |
|  |  | AN/OCD | 0.063 | 0.028 | 2.290 | **0.022^*^** |
| Restraint at age 14 | 4074 | AN | 0.036 | 0.017 | 2.072 | **0.038^*^** |
|  |  | OCD | 0.003 | 0.017 | 0.177 | 0.859 |
|  |  | AN/OCD | 0.037 | 0.017 | 2.097 | **0.036^*^** |
| Emotional eating at age 14 | 3927 | AN | 0.117 | 0.086 | 1.351 | 0.177 |
|  |  | OCD | 0.007 | 0.085 | 0.081 | 0.935 |
|  |  | AN/OCD | 0.080 | 0.086 | 0.929 | 0.353 |
| External eating at age 14 | 3608 | AN | 0.068 | 0.056 | 1.218 | 0.223 |
|  |  | OCD | 0.049 | 0.055 | 0.883 | 0.377 |
|  |  | AN/OCD | 0.045 | 0.056 | 0.805 | 0.421 |
| Thin ideal internalization at age 14 | 4053 | AN | 0.075 | 0.042 | 1.778 | 0.076 |
|  |  | OCD | 0.071 | 0.041 | 1.732 | 0.083 |
|  |  | AN/OCD | 0.079 | 0.042 | 1.875 | 0.061 |
| Body dissatisfaction at age 14 | 4169 | AN | 0.261 | 0.117 | 2.228 | **0.026^*^** |
|  |  | OCD | 0.122 | 0.116 | 1.057 | 0.291 |
|  |  | AN/OCD | 0.265 | 0.117 | 2.265 | **0.024^*^** |
| Weight and shape concern at age 14 | 4164 | AN | 0.033 | 0.027 | 1.216 | 0.224 |
|  |  | OCD | 0.009 | 0.027 | 0.313 | 0.755 |
|  |  | AN/OCD | 0.039 | 0.027 | 1.412 | 0.158 |
| AN at age 14^b^ | 106 cases,  4129 controls | AN | 0.150 | 0.100 | 1.502 | 0.133 |
|  |  | OCD | -0.045 | 0.098 | -0.459 | 0.646 |
|  |  | AN/OCD | 0.113 | 0.100 | 1.135 | 0.256 |
| Bulimia nervosa or subthreshold bulimia nervosa at age 14^b^ | 63 cases,  4172 controls | AN | 0.050 | 0.129 | 0.385 | 0.700 |
|  |  | OCD | -0.110 | 0.126 | -0.868 | 0.386 |
|  |  | AN/OCD | 0.032 | 0.129 | 0.248 | 0.804 |
| Binge-eating disorder or subthreshold binge-eating disorder at age 14^b,c^ | 25 cases,  4210 controls | AN | -- | -- | -- | -- |
|  |  | OCD | -- | -- | -- | -- |
|  |  | AN/OCD | -- | -- | -- | -- |
| Eating disorders not otherwise specified or purging disorder at age 14^b^ | 593 cases,  3642 controls | AN | 0.111 | 0.046 | 2.446 | **0.015^*^** |
|  |  | OCD | 0.001 | 0.044 | 0.025 | 0.980 |
|  |  | AN/OCD | 0.112 | 0.045 | 2.480 | **0.013^*^** |
| Any threshold/subthreshold eating disorder at age 14^b^ | 787 cases,  3448 controls | AN | 0.115 | 0.041 | 2.813 | **0.005^*^** |
|  |  | OCD | -0.009 | 0.040 | -0.220 | 0.826 |
|  |  | AN/OCD | 0.112 | 0.041 | 2.757 | **0.006^*^** |
| Fasting at age 14 | 3932 | AN | 0.006 | 0.004 | 1.569 | 0.117 |
|  |  | OCD | -0.001 | 0.004 | -0.263 | 0.793 |
|  |  | AN/OCD | 0.008 | 0.004 | 2.146 | **0.032^*^** |
| Purging at age 14 | 4132 | AN | 0.002 | 0.004 | 0.436 | 0.663 |
|  |  | OCD | -0.006 | 0.004 | -1.536 | 0.125 |
|  |  | AN/OCD | 0.002 | 0.004 | 0.491 | 0.624 |
| Binge eating at age 14 | 4167 | AN | < 0.001 | 0.006 | 0.067 | 0.947 |
|  |  | OCD | 0.006 | 0.006 | 0.902 | 0.367 |
|  |  | AN/OCD | 0.003 | 0.006 | 0.459 | 0.646 |
| Compulsive exercise at age 14 | 4027 | AN | 0.025 | 0.010 | 2.537 | **0.011^*^** |
|  |  | OCD | 0.007 | 0.010 | 0.711 | 0.477 |
|  |  | AN/OCD | 0.023 | 0.010 | 2.348 | **0.019^*^** |
| AN at age 16^b^ | 71 cases,  3511 controls | AN | -0.096 | 0.121 | -0.789 | 0.430 |
|  |  | OCD | 0.084 | 0.119 | 0.710 | 0.478 |
|  |  | AN/OCD | -0.134 | 0.120 | -1.119 | 0.263 |
| Bulimia nervosa at age 16^b^ | 146 cases,  3436 controls | AN | 0.135 | 0.086 | 1.575 | 0.115 |
|  |  | OCD | -0.008 | 0.084 | -0.090 | 0.929 |
|  |  | AN/OCD | 0.124 | 0.085 | 1.456 | 0.145 |
| Binge-eating disorder at age 16^b^ | 58 cases,  3524 controls | AN | -0.084 | 0.133 | -0.627 | 0.531 |
|  |  | OCD | 0.129 | 0.131 | 0.979 | 0.328 |
|  |  | AN/OCD | -0.036 | 0.132 | -0.275 | 0.783 |
| Eating disorders not otherwise specified or purging disorder at age 16^b^ | 1079 cases,  2503 controls | AN | 0.042 | 0.038 | 1.108 | 0.268 |
|  |  | OCD | 0.037 | 0.038 | 0.987 | 0.324 |
|  |  | AN/OCD | 0.033 | 0.038 | 0.865 | 0.387 |
| Any threshold/subthreshold eating disorder at age 16^b^ | 1354 cases,  2228 controls | AN | 0.049 | 0.037 | 1.336 | 0.181 |
|  |  | OCD | 0.051 | 0.036 | 1.398 | 0.162 |
|  |  | AN/OCD | 0.038 | 0.037 | 1.046 | 0.295 |
| Fasting at age 16 | 3379 | AN | -0.002 | 0.011 | -0.193 | 0.847 |
|  |  | OCD | 0.004 | 0.011 | 0.343 | 0.732 |
|  |  | AN/OCD | -0.007 | 0.011 | -0.614 | 0.539 |
| Purging at age 16 | 3402 | AN | 0.009 | 0.008 | 1.042 | 0.298 |
|  |  | OCD | 0.008 | 0.008 | 1.009 | 0.313 |
|  |  | AN/OCD | 0.008 | 0.008 | 0.992 | 0.321 |
| Binge eating at age 16 | 2929 | AN | 0.015 | 0.012 | 1.262 | 0.207 |
|  |  | OCD | 0.013 | 0.012 | 1.055 | 0.292 |
|  |  | AN/OCD | 0.018 | 0.012 | 1.478 | 0.140 |
| Compulsive exercise at age 16 | 3186 | AN | 0.023 | 0.017 | 1.369 | 0.171 |
|  |  | OCD | 0.038 | 0.016 | 2.371 | **0.018^*^** |
|  |  | AN/OCD | 0.016 | 0.017 | 0.969 | 0.333 |
| **Obsessive-Compulsive Disorder Symptom Dimensions and Diagnosis** | | | | | | |
| **Phenotype** | **Sample size** | **PGS** | ***ß*** | ***SE*** | **Test statistic^d^** | ***P*** |
| OCD at age 10^b,c^ | 18 cases,  5198 controls | AN | -- | -- | -- | -- |
|  |  | OCD | -- | -- | -- | -- |
|  |  | AN/OCD | -- | -- | -- | -- |
| OCD latent factor – symmetry, checking at age 10 | 5197 | AN | 0.011 | 0.007 | 1.581 | 0.114 |
|  |  | OCD | 0.002 | 0.007 | 0.217 | 0.828 |
|  |  | AN/OCD | 0.010 | 0.007 | 1.418 | 0.156 |
| OCD latent factor – dirt/germs at age 10 | 5197 | AN | 0.008 | 0.006 | 1.327 | 0.185 |
|  |  | OCD | 0.006 | 0.006 | 0.979 | 0.328 |
|  |  | AN/OCD | 0.008 | 0.006 | 1.254 | 0.210 |
| OCD at age 13^b,c^ | 10 cases  4841 controls | AN | -- | -- | -- | -- |
|  |  | OCD | -- | -- | -- | -- |
|  |  | AN/OCD | -- | -- | -- | -- |
| OCD latent factor – symmetry, checking at age 13 | 4832 | AN | -0.003 | 0.010 | -0.312 | 0.755 |
|  |  | OCD | 0.001 | 0.010 | 0.098 | 0.922 |
|  |  | AN/OCD | -0.002 | 0.010 | -0.154 | 0.878 |
| OCD latent factor – dirt/germs at age 13 | 4832 | AN | -0.001 | 0.009 | -0.104 | 0.918 |
|  |  | OCD | -0.016 | 0.009 | -1.732 | 0.083 |
|  |  | AN/OCD | -0.001 | 0.009 | -0.088 | 0.930 |
| **Anxiety Symptom Dimensions and Diagnoses** | | | | | | |
| **Phenotype** | **Sample size** | **PGS** | ***ß*** | ***SE*** | **Test statistic^d^** | ***P*** |
| Separation anxiety at age 7^b^ | 148 cases,  5214 controls | AN | 0.226 | 0.084 | 2.680 | **0.007^*^** |
|  |  | OCD | 0.019 | 0.083 | 0.233 | 0.816 |
|  |  | AN/OCD | 0.255 | 0.085 | 3.020 | **0.003^*^** |
| Specific phobia at age 7^b^ | 104 cases,  5265 controls | AN | 0.177 | 0.100 | 1.769 | 0.077 |
|  |  | OCD | 0.052 | 0.099 | 0.528 | 0.598 |
|  |  | AN/OCD | 0.200 | 0.100 | 1.955 | 0.051 |
| Latent factor – physical anxiety at age 10 | 5197 | AN | 0.023 | 0.010 | 2.235 | **0.026^*^** |
|  |  | OCD | < 0.001 | 0.010 | 0.044 | 0.965 |
|  |  | AN/OCD | 0.015 | 0.010 | 1.431 | 0.152 |
| Latent factor – worrying at age 10 | 5197 | AN | 0.019 | 0.010 | 1.888 | 0.059 |
|  |  | OCD | 0.007 | 0.010 | 0.664 | 0.507 |
|  |  | AN/OCD | 0.012 | 0.010 | 1.234 | 0.217 |
| Latent factor – social phobia at age 10 | 5197 | AN | 0.011 | 0.009 | 1.279 | 0.201 |
|  |  | OCD | -0.006 | 0.009 | -0.683 | 0.495 |
|  |  | AN/OCD | 0.006 | 0.009 | 0.715 | 0.475 |
| Social phobia at age 13^b^ | 55 cases,  4795 controls | AN | 0.032 | 0.137 | 0.236 | 0.813 |
|  |  | OCD | 0.220 | 0.136 | 1.611 | 0.107 |
|  |  | AN/OCD | 0.025 | 0.137 | 0.180 | 0.857 |
| Generalized anxiety disorder at age 13^b^ | 154 cases,  4687 controls | AN | 0.114 | 0.083 | 1.366 | 0.172 |
|  |  | OCD | 0.148 | 0.082 | 1.802 | 0.072 |
|  |  | AN/OCD | 0.133 | 0.083 | 1.611 | 0.107 |
| Latent factor – physical anxiety at age 13 | 4832 | AN | -0.007 | 0.010 | -0.729 | 0.466 |
|  |  | OCD | < 0.001 | 0.010 | 0.017 | 0.987 |
|  |  | AN/OCD | -0.007 | 0.010 | -0.687 | 0.492 |
| Latent factor – worrying at age 13 | 4832 | AN | 0.015 | 0.011 | 1.363 | 0.173 |
|  |  | OCD | 0.002 | 0.011 | 0.167 | 0.867 |
|  |  | AN/OCD | 0.015 | 0.0112 | 1.364 | 0.172 |
| Latent factor – social phobia at age 13 | 4832 | AN | 0.025 | 0.011 | 2.359 | **0.018^*^** |
|  |  | OCD | < 0.001 | 0.011 | 0.074 | 0.941 |
|  |  | AN/OCD | 0.022 | 0.011 | 2.080 | **0.038^*^** |
| Generalized anxiety disorder at age 15^b^ | 196 cases,  3712 controls | AN | 0.038 | 0.075 | 0.508 | 0.611 |
|  |  | OCD | 0.006 | 0.074 | 0.074 | 0.941 |
|  |  | AN/OCD | 0.024 | 0.075 | 0.318 | 0.750 |

^a^ Genomic principal components 1-5 were used as covariates to account for population stratification.

^b^ Binary phenotype

^c^ Due to insufficient statistical power, any binary measure with less than 50 cases is not included in the final analysis.

^d^ We report *t*-values for continuous phenotypes and *z*-values for binary phenotypes.

^*^ (also bolded) Statistically significant at *P* < 0.05.

Abbreviations: PGS=polygenic score; *ß*=standardized beta regression coefficient; *SE*=standard error; AN=anorexia nervosa; OCD=obsessive-compulsive disorder; AN/OCD=anorexia nervosa/obsessive-compulsive transdiagnostic phenotype.

**Table S4. Prediction of eating disorder, obsessive-compulsive disorder, and anxiety symptom dimensions and diagnoses using polygenic scores in all participants, without sex as a covariate^a^**

| **Eating Disorder Symptom Dimensions and Diagnoses** | | | | | | |
| --- | --- | --- | --- | --- | --- | --- |
| **Phenotype** | **Sample size** | **PGS** | ***ß*** | ***SE*** | **Test statistic^d^** | ***P*** |
| Body image distortion at age 10 | 4758 | AN | 0.001 | 0.010 | 0.047 | 0.962 |
|  |  | OCD | -0.008 | 0.010 | -0.791 | 0.429 |
|  |  | AN/OCD | 0.010 | 0.010 | 0.196 | 0.844 |
| Fear of weight gain at age 14 | 4127 | AN | 0.011 | 0.011 | 0.994 | 0.320 |
|  |  | OCD | 0.004 | 0.011 | 0.353 | 0.724 |
|  |  | AN/OCD | 0.016 | 0.011 | 1.495 | 0.135 |
| Pressure to lose weight at age 14 | 4116 | AN | 0.057 | 0.028 | 1.995 | **0.046^*^** |
|  |  | OCD | 0.003 | 0.028 | 0.104 | 0.917 |
|  |  | AN/OCD | 0.057 | 0.028 | 2.028 | **0.043^*^** |
| Restraint at age 14 | 4074 | AN | 0.031 | 0.018 | 1.736 | 0.083 |
|  |  | OCD | 0.001 | 0.018 | 0.036 | 0.971 |
|  |  | AN/OCD | 0.033 | 0.018 | 1.822 | 0.069 |
| Emotional eating at age 14 | 3927 | AN | 0.084 | 0.090 | 0.935 | 0.350 |
|  |  | OCD | -0.008 | 0.089 | -0.089 | 0.929 |
|  |  | AN/OCD | 0.056 | 0.090 | 0.618 | 0.537 |
| External eating at age 14 | 3608 | AN | 0.069 | 0.056 | 1.230 | 0.218 |
|  |  | OCD | 0.049 | 0.055 | 0.892 | 0.372 |
|  |  | AN/OCD | 0.045 | 0.056 | 0.814 | 0.416 |
| Thin ideal internalization at age 14 | 4053 | AN | 0.086 | 0.043 | 1.987 | **0.047^*^** |
|  |  | OCD | 0.077 | 0.042 | 1.811 | 0.070 |
|  |  | AN/OCD | 0.087 | 0.043 | 2.027 | **0.043^*^** |
| Body dissatisfaction at age 14 | 4169 | AN | 0.228 | 0.121 | 1.876 | 0.061 |
|  |  | OCD | 0.108 | 0.120 | 0.903 | 0.367 |
|  |  | AN/OCD | 0.240 | 0.121 | 1.983 | **0.047^*^** |
| Weight and shape concern at age 14 | 4164 | AN | 0.023 | 0.029 | 0.793 | 0.428 |
|  |  | OCD | 0.004 | 0.029 | 0.152 | 0.879 |
|  |  | AN/OCD | 0.030 | 0.029 | 1.055 | 0.290 |
| AN at age 14^b^ | 106 cases,  4129 controls | AN | 0.141 | 0.099 | 1.417 | 0.157 |
|  |  | OCD | -0.051 | 0.098 | -0.516 | 0.606 |
|  |  | AN/OCD | 0.107 | 0.099 | 1.075 | 0.282 |
| Bulimia nervosa or subthreshold bulimia nervosa at age 14^b^ | 63 cases,  4172 controls | AN | 0.043 | 0.129 | 0.333 | 0.739 |
|  |  | OCD | -0.115 | 0.127 | -0.905 | 0.366 |
|  |  | AN/OCD | 0.026 | 0.129 | 0.204 | 0.838 |
| Binge-eating disorder or subthreshold binge-eating disorder at age 14^b,c^ | 25 cases,  4210 controls | AN | -- | -- | -- | -- |
|  |  | OCD | -- | -- | -- | -- |
|  |  | AN/OCD | -- | -- | -- | -- |
| Eating disorders not otherwise specified or purging disorder at age 14^b^ | 593 cases,  3642 controls | AN | 0.101 | 0.045 | 2.246 | **0.025^*^** |
|  |  | OCD | -0.002 | 0.044 | -0.048 | 0.962 |
|  |  | AN/OCD | 0.104 | 0.045 | 2.323 | **0.020^*^** |
| Any threshold/subthreshold eating disorder at age 14^b^ | 787 cases,  3448 controls | AN | 0.103 | 0.040 | 2.564 | **0.010^*^** |
|  |  | OCD | -0.013 | 0.039 | -0.318 | 0.751 |
|  |  | AN/OCD | 0.103 | 0.040 | 2.557 | **0.011^*^** |
| Fasting at age 14 | 3932 | AN | 0.006 | 0.004 | 1.401 | 0.161 |
|  |  | OCD | -0.001 | 0.004 | -0.350 | 0.726 |
|  |  | AN/OCD | 0.008 | 0.004 | 2.012 | **0.044^*^** |
| Purging at age 14 | 4132 | AN | 0.002 | 0.004 | 0.366 | 0.715 |
|  |  | OCD | -0.006 | 0.004 | -1.575 | 0.115 |
|  |  | AN/OCD | 0.002 | 0.004 | 0.437 | 0.662 |
| Binge eating at age 14 | 4167 | AN | < 0.001 | 0.006 | -0.001 | 0.999 |
|  |  | OCD | 0.006 | 0.006 | 0.865 | 0.387 |
|  |  | AN/OCD | 0.003 | 0.006 | 0.406 | 0.685 |
| Compulsive exercise at age 14 | 4027 | AN | 0.023 | 0.010 | 2.259 | **0.024^*^** |
|  |  | OCD | 0.006 | 0.010 | 0.598 | 0.550 |
|  |  | AN/OCD | 0.021 | 0.010 | 2.128 | **0.033^*^** |
| AN at age 16^b^ | 71 cases,  3511 controls | AN | -0.098 | 0.121 | -0.812 | 0.417 |
|  |  | OCD | 0.083 | 0.119 | 0.693 | 0.488 |
|  |  | AN/OCD | -0.138 | 0.120 | -1.146 | 0.252 |
| Bulimia nervosa at age 16^b^ | 146 cases,  3436 controls | AN | 0.126 | 0.085 | 1.478 | 0.139 |
|  |  | OCD | -0.006 | 0.084 | -0.069 | 0.945 |
|  |  | AN/OCD | 0.117 | 0.085 | 1.373 | 0.170 |
| Binge-eating disorder at age 16^b^ | 58 cases,  3524 controls | AN | -0.092 | 0.133 | -0.694 | 0.488 |
|  |  | OCD | 0.130 | 0.132 | 0.984 | 0.320 |
|  |  | AN/OCD | -0.046 | 0.133 | -0.346 | 0.729 |
| Eating disorders not otherwise specified or purging disorder at age 16^b^ | 1079 cases,  2503 controls | AN | 0.030 | 0.037 | 0.830 | 0.406 |
|  |  | OCD | 0.035 | 0.036 | 0.971 | 0.331 |
|  |  | AN/OCD | 0.022 | 0.037 | 0.611 | 0.541 |
| Any threshold/subthreshold eating disorder at age 16^b^ | 1354 cases,  2228 controls | AN | 0.034 | 0.035 | 0.974 | 0.330 |
|  |  | OCD | 0.046 | 0.034 | 1.346 | 0.178 |
|  |  | AN/OCD | 0.025 | 0.035 | 0.717 | 0.473 |
| Fasting at age 16 | 3379 | AN | -0.005 | 0.011 | -0.395 | 0.693 |
|  |  | OCD | 0.003 | 0.011 | 0.294 | 0.769 |
|  |  | AN/OCD | -0.009 | 0.011 | -0.792 | 0.428 |
| Purging at age 16 | 3402 | AN | 0.008 | 0.009 | 0.890 | 0.374 |
|  |  | OCD | 0.008 | 0.008 | 0.969 | 0.332 |
|  |  | AN/OCD | 0.007 | 0.009 | 0.848 | 0.397 |
| Binge eating at age 16 | 2929 | AN | 0.013 | 0.012 | 1.103 | 0.270 |
|  |  | OCD | 0.012 | 0.012 | 1.015 | 0.310 |
|  |  | AN/OCD | 0.016 | 0.012 | 1.344 | 0.179 |
| Compulsive exercise at age 16 | 3186 | AN | 0.018 | 0.017 | 1.046 | 0.295 |
|  |  | OCD | 0.037 | 0.017 | 2.208 | **0.027^*^** |
|  |  | AN/OCD | 0.012 | 0.017 | 0.688 | 0.491 |
| **Obsessive-Compulsive Disorder Symptom Dimensions and Diagnosis** | | | | | | |
| **Phenotype** | **Sample size** | **PGS** | ***ß*** | ***SE*** | **Test statistic^d^** | ***P*** |
| OCD at age 10^b,c^ | 18 cases,  5198 controls | AN | -- | -- | -- | -- |
|  |  | OCD | -- | -- | -- | -- |
|  |  | AN/OCD | -- | -- | -- | -- |
| OCD latent factor – symmetry, checking at age 10 | 5197 | AN | 0.011 | 0.007 | 1.583 | 0.113 |
|  |  | OCD | 0.002 | 0.007 | 0.221 | 0.825 |
|  |  | AN/OCD | 0.010 | 0.007 | 1.419 | 0.156 |
| OCD latent factor – dirt/germs at age 10 | 5197 | AN | 0.008 | 0.006 | 1.328 | 0.184 |
|  |  | OCD | 0.006 | 0.006 | 0.981 | 0.327 |
|  |  | AN/OCD | 0.008 | 0.006 | 1.254 | 0.210 |
| OCD at age 13^b,c^ | 10 cases  4841 controls | AN | -- | -- | -- | -- |
|  |  | OCD | -- | -- | -- | -- |
|  |  | AN/OCD | -- | -- | -- | -- |
| OCD latent factor – symmetry, checking at age 13 | 4832 | AN | -0.004 | 0.011 | -0.413 | 0.680 |
|  |  | OCD | 0.002 | 0.011 | 0.189 | 0.850 |
|  |  | AN/OCD | -0.002 | 0.011 | -0.197 | 0.844 |
| OCD latent factor – dirt/germs at age 13 | 4832 | AN | -0.001 | 0.009 | -0.133 | 0.894 |
|  |  | OCD | -0.016 | 0.009 | -1.702 | 0.089 |
|  |  | AN/OCD | -0.001 | 0.009 | -0.101 | 0.920 |
| **Anxiety Symptom Dimensions and Diagnoses** | | | | | | |
| **Phenotype** | **Sample size** | **PGS** | ***ß*** | ***SE*** | **Test statistic^d^** | ***P*** |
| Separation anxiety at age 7^b^ | 148 cases,  5214 controls | AN | 0.226 | 0.084 | 2.679 | **0.007^*^** |
|  |  | OCD | 0.019 | 0.083 | 0.230 | 0.818 |
|  |  | AN/OCD | 0.255 | 0.084 | 3.021 | **0.003^*^** |
| Specific phobia at age 7^b^ | 104 cases,  5265 controls | AN | 0.177 | 0.100 | 1.767 | 0.077 |
|  |  | OCD | 0.052 | 0.099 | 0.527 | 0.598 |
|  |  | AN/OCD | 0.195 | 0.100 | 1.952 | 0.051 |
| Latent factor – physical anxiety at age 10 | 5197 | AN | 0.023 | 0.010 | 2.230 | **0.026^*^** |
|  |  | OCD | < 0.001 | 0.010 | 0.038 | 0.970 |
|  |  | AN/OCD | 0.015 | 0.010 | 1.429 | 0.153 |
| Latent factor – worrying at age 10 | 5197 | AN | 0.019 | 0.001 | 1.873 | 0.061 |
|  |  | OCD | 0.006 | 0.010 | 0.649 | 0.517 |
|  |  | AN/OCD | 0.012 | 0.010 | 1.227 | 0.220 |
| Latent factor – social phobia at age 10 | 5197 | AN | 0.011 | 0.009 | 1.280 | 0.201 |
|  |  | OCD | -0.006 | 0.009 | -0.681 | 0.496 |
|  |  | AN/OCD | 0.006 | 0.009 | 0.716 | 0.474 |
| Social phobia at age 13^b^ | 55 cases,  4795 controls | AN | 0.032 | 0.137 | 0.232 | 0.816 |
|  |  | OCD | 0.221 | 0.136 | 1.619 | 0.106 |
|  |  | AN/OCD | 0.025 | 0.137 | 0.179 | 0.858 |
| Generalized anxiety disorder at age 13^b^ | 154 cases,  4687 controls | AN | 0.111 | 0.083 | 1.343 | 0.179 |
|  |  | OCD | 0.149 | 0.082 | 1.820 | 0.069 |
|  |  | AN/OCD | 0.133 | 0.083 | 1.606 | 0.108 |
| Latent factor – physical anxiety at age 13 | 4832 | AN | -0.007 | 0.010 | -0.727 | 0.467 |
|  |  | OCD | < 0.001 | 0.010 | 0.015 | 0.988 |
|  |  | AN/OCD | -0.007 | 0.010 | -0.686 | 0.493 |
| Latent factor – worrying at age 13 | 4832 | AN | 0.015 | 0.011 | 1.292 | 0.196 |
|  |  | OCD | 0.002 | 0.011 | 0.213 | 0.831 |
|  |  | AN/OCD | 0.015 | 0.011 | 1.327 | 0.185 |
| Latent factor – social phobia at age 13 | 4832 | AN | 0.025 | 0.011 | 2.266 | **0.024^*^** |
|  |  | OCD | 0.001 | 0.011 | 0.127 | 0.899 |
|  |  | AN/OCD | 0.022 | 0.011 | 2.027 | **0.043^*^** |
| Generalized anxiety disorder at age 15^b^ | 196 cases,  3712 controls | AN | 0.023 | 0.074 | 0.316 | 0.752 |
|  |  | OCD | 0.010 | 0.073 | 0.138 | 0.891 |
|  |  | AN/OCD | 0.014 | 0.074 | 0.185 | 0.853 |

^a^ Genomic principal components 1-5 were used as covariates to account for population stratification.

^b^ Binary phenotype

^c^ Due to insufficient statistical power, any binary measure with less than 50 cases is not included in the final analysis.

^d^ We report *t*-values for continuous phenotypes and *z*-values for binary phenotypes.

^*^ (also bolded) Statistically significant at *P* < 0.05.

Abbreviations: PGS=polygenic score; *ß*=standardized beta regression coefficient; *SE*=standard error; AN=anorexia nervosa; OCD=obsessive-compulsive disorder; AN/OCD=anorexia nervosa/obsessive-compulsive transdiagnostic phenotype.

**Figure S1. (a) The quantile-quantile plot and (b) the Manhattan plot for the anorexia nervosa/obsessive-compulsive disorder transdiagnostic genome-wide association meta-analysis (19,680 cases and 62,556 controls).**

**CONSORTIA CO-AUTHORS**

**Anorexia Nervosa Genetics Initiative**

Jessica H. Baker, Andrew W. Bergen, Andreas Birgegärd, Joseph M. Boden, Harry Brandt, Cynthia M. Bulik, Steven Crawford, Laramie E. Duncan, Scott Gordon, Jakob Grove, Katherine A. Halmi, Anjali K. Henders, L. John Horwood, Craig Johnson, Jennifer Jordan, Anders Juréus, Allan S. Kaplan, Walter Kaye, Martin Kennedy, Katherine M. Kirk, Mikael Landén, Janne T. Larsen, Virpi M. Leppä, Paul Lichtenstein, Nicholas G. Martin, Manuel Mattheisen, James Mitchell, Grant W. Montgomery, Preben Bo Mortensen, Melissa A. Munn-Chernoff, Claes Norring, Catherine M. Olsen, Richard Parker, John F. Pearson, Nancy L. Pedersen, Liselotte Petersen, Michael Strober, Patrick F. Sullivan, Laura M. Thornton, Tracey D. Wade, Hunna J. Watson, Thomas Werge, David C. Whiteman, D. Blake Woodside, Zeynep Yilmaz

**Eating Disorders Working Group of the PGC**

Hunna J. Watson, Zeynep Yilmaz, Laura M. Thornton, Christopher Hübel, Jonathan R. I. Coleman, Héléna A. Gaspar, Julien Bryois, Anke Hinney, Virpi M. Leppä, Manuel Mattheisen, Sarah E. Medland, Stephan Ripke, Shuyang Yao, Paola Giusti-Rodríguez, Ken B. Hanscombe, Kirstin L. Purves, Roger A. H. Adan, Lars Alfredsson, Tetsuya Ando, Ole A. Andreassen, Jessica H. Baker, Wade H. Berrettini, Ilka Boehm, Claudette Boni, Vesna Boraska Perica, Katharina Buehren, Roland Burghardt, Matteo Cassina, Sven Cichon, Maurizio Clementi, Roger D. Cone, Philippe Courtet, Scott Crow, James J. Crowley, Unna N. Danner, Oliver S. P. Davis, Martina de Zwaan, George Dedoussis, Daniela Degortes, Janiece E. DeSocio, Danielle M. Dick, Dimitris Dikeos, Christian Dina, Monika Dmitrzak-Weglarz, Elisa Docampo, Laramie E. Duncan, Karin Egberts, Stefan Ehrlich, Geòrgia Escaramís, Tõnu Esko, Xavier Estivill, Anne Farmer, Angela Favaro, Fernando Fernández-Aranda, Manfred M. Fichter, Krista Fischer, Manuel Föcker, Lenka Foretova, Andreas J. Forstner, Monica Forzan, Christopher S. Franklin, Steven Gallinger, Ina Giegling, Johanna Giuranna, Fragiskos Gonidakis, Philip Gorwood, Monica Gratacos Mayora, Sébastien Guillaume, Yiran Guo, Hakon Hakonarson, Konstantinos Hatzikotoulas, Joanna Hauser, Johannes Hebebrand, Sietske G. Helder, Stefan Herms, Beate Herpertz-Dahlmann, Wolfgang Herzog, Laura M. Huckins, James I. Hudson, Hartmut Imgart, Hidetoshi Inoko, Vladimir Janout, Susana Jiménez-Murcia, Antonio Julià, Gursharan Kalsi, Deborah Kaminská, Jaakko Kaprio, Leila Karhunen, Andreas Karwautz, Martien J. H. Kas, James L. Kennedy, Anna Keski-Rahkonen, Kirsty Kiezebrink, Youl-Ri Kim, Lars Klareskog, Kelly L. Klump, Gun Peggy S. Knudsen, Maria C. La Via1, Stephanie Le Hellard, Robert D. Levitan, Dong Li, Lisa Lilenfeld, Bochao Danae Lin, Jolanta Lissowska, Jurjen Luykx, Pierre J. Magistretti, Mario Maj, Katrin Mannik, Sara Marsal,

Christian R. Marshall, Morten Mattingsdal, Sara McDevitt, Peter McGuffin, Andres Metspalu, Ingrid Meulenbelt, Nadia Micali, Karen Mitchell, Alessio Maria Monteleone, Palmiero Monteleone, Melissa A. Munn-Chernoff, Benedetta Nacmias, Marie Navratilova, Ioanna Ntalla, Julie K. O’Toole, Roel A. Ophoff, Leonid Padyukov, Aarno Palotie, Jacques Pantel, Hana Papezova, Dalila Pinto, Raquel Rabionet, Anu Raevuori, Nicolas Ramoz, Ted Reichborn-Kjennerud, Valdo Ricca, Samuli Ripatti, Franziska Ritschel, Marion Roberts, Alessandro Rotondo, Dan Rujescu, Filip Rybakowski, Paolo Santonastaso, André Scherag, Stephen W. Scherer, Ulrike Schmidt, Nicholas J. Schork, Alexandra Schosser, Jochen Seitz, Lenka Slachtova, P. Eline Slagboom, Margarita C. T. Slof-Op ‘t Landt, Agnieszka Slopien, Sandro Sorbi, Beata Świątkowska, Jin P. Szatkiewicz, Ioanna Tachmazidou, Elena Tenconi, Alfonso Tortorella, Federica Tozzi, Janet Treasure, Artemis Tsitsika, Marta Tyszkiewicz-Nwafor, Konstantinos Tziouvas, Annemarie A. van Elburg, Eric F. van Furth, Gudrun Wagner, Esther Walton, Elisabeth Widen, Eleftheria Zeggini, Stephanie Zerwas, Stephan Zipfel, Andrew W. Bergen, Joseph M. Boden, Harry Brandt, Steven Crawford, Katherine A. Halmi, L. John Horwood, Craig Johnson, Allan S. Kaplan, Walter H. Kaye, James E. Mitchell, Catherine M. Olsen, John F. Pearson, Nancy L. Pedersen, Michael Strober, Thomas Werge, David C. Whiteman, D. Blake Woodside, Garret D. Stuber, Scott Gordon, Jakob Grove, Anjali K. Henders, Anders Juréus, Katherine M. Kirk, Janne T. Larsen, Richard Parker, Liselotte Petersen, Jennifer Jordan, Martin Kennedy, Grant W. Montgomery, Tracey D. Wade, Andreas Birgegård, Paul Lichtenstein, Claes Norring, Mikael Landén, Nicholas G. Martin, Preben Bo Mortensen, Patrick F. Sullivan, Gerome Breen, Cynthia M. Bulik.

**Obsessive Compulsive Disorder Working Group of the PGC**

Paul D. Arnold, Kathleen D. Askland, Cristina Barlassina, Laura Bellodi, O. J. Bienvenu, Donald Black, Michael Bloch, Helena Brentani, Christie L. Burton, Beatriz Camarena, Carolina Cappi, Danielle Cath, Maria Cavallini, David Conti, Edwin Cook, Vladimir Coric, Bernadette A. Cullen, Danielle Cusi, Lea K. Davis, Richard Delorme, Damiaan Denys, Eske Derks, Valsamma Eapen, Christopher Edlund, Lauren Erdman, Peter Falkai, Martijn Figee, Abigail J. Fyer, Daniel A Geller, Fernando S. Goes, Hans Grabe, Marcos A. Grados, Benjamin D. Greenberg, Edna Grünblatt, Wei Guo, Gregory L. Hanna, Sian Hemmings, Ana G. Hounie, Michael Jenicke, Clare Keenan, James Kennedy, Ekaterina A. Khramtsova, Anuar Konkashbaev, James A. Knowles, Janice Krasnow, Cristophe Lange, Nuria Lanzagorta, Marion Leboyer, Leonhard Lennertz, Bingbin Li, K-Y Liang, Christine Lochner, Fabio Macciardi, Brion Maher, Wolfgang Maier, Maurizio Marconi, Carol A. Mathews, Manuel Mattheisen, James T. McCracken, Nicole C. McLaughlin, Euripedes C. Miguel, Rainald Moessner, Dennis L. Murphy, Benjamin Neale, Gerald Nestadt, Paul Nestadt, Humberto Nicolini, Ericka Nurmi, Lisa Osiecki, David L. Pauls, John Piacentini, Danielle Posthuma, Ann E. Pulver, H-D Qin, Steven A. Rasmussen, Scott Rauch, Margaret A. Richter, Mark A. Riddle, Stephan Ripke, Stephan Ruhrmann, Aline S. Sampaio, Jack F. Samuels, Jeremiah M. Scharf, Yin Yao Shugart, Jan Smit, Daniel Stein, S. Evelyn Stewart, Maurizio Turiel, Homero Vallada, Jeremy Veenstra-VanderWeele, Michael Wagner, Susanne Walitza, Y. Wang, Jens Wendland, Nienke Vulink, Dongmei Yu, Gwyneth Zai.
